# Hesperetin activates CISD2 to attenuate senescence in human keratinocytes from an older person and rejuvenates naturally aged skin in mice

**DOI:** 10.1186/s12929-024-01005-w

**Published:** 2024-01-23

**Authors:** Zhao-Qing Shen, Cheng-Yen Chang, Chi-Hsiao Yeh, Chung-Kuang Lu, Hao-Chih Hung, Tai-Wen Wang, Kuan-Sheng Wu, Chien-Yi Tung, Ting-Fen Tsai

**Affiliations:** 1 Department of Life Sciences and Institute of Genome Sciences, National Yang Ming Chiao Tung University, No. 155, Sec. 2, Li-Nong Street, Peitou, Taipei 112 Taiwan; 2 Department of Thoracic and Cardiovascular Surgery, Chang Gung Memorial Hospital, Linkou, Taoyuan Taiwan; 3 College of Medicine, Chang Gung University, Taoyuan, Taiwan; 4 National Research Institute of Chinese Medicine, Taipei, Taiwan; 5 Genomics Center for Clinical and Biotechnological Applications, Cancer and Immunology Research Center, National Yang Ming Chiao Tung University, Taipei, Taiwan; 6 Center for Healthy Longevity and Aging Sciences, National Yang Ming Chiao Tung University, Taipei, Taiwan; 7 Institute of Molecular and Genomic Medicine, National Health Research Institutes, Zhunan, Taiwan

**Keywords:** CISD2, Skin aging, Skin rejuvenation, Keratinocytes, Cellular senescence, CISD2 activator, Hesperetin, Mitochondrial function

## Abstract

**Background:**

CDGSH iron-sulfur domain-containing protein 2 (CISD2), a pro-longevity gene, mediates healthspan in mammals. CISD2 is down-regulated during aging. Furthermore, a persistently high level of CISD2 promotes longevity and ameliorates an age-related skin phenotype in transgenic mice. Here we translate the genetic evidence into a pharmaceutical application using a potent CISD2 activator, hesperetin, which enhances CISD2 expression in HEK001 human keratinocytes from an older person. We also treated naturally aged mice in order to study the activator’s anti-aging efficacy.

**Methods:**

We studied the biological effects of hesperetin on aging skin using, firstly, a cell-based platform, namely a HEK001 human keratinocyte cell line established from an older person. Secondly, we used a mouse model, namely old mice at 21-month old. In the latter case, we investigate the anti-aging efficacy of hesperetin on ultraviolet B (UVB)-induced photoaging and naturally aged skin. Furthermore, to identify the underlying mechanisms and potential biological pathways involved in this process we carried out transcriptomic analysis. Finally, CISD2 knockdown HEK001 keratinocytes and Cisd2 knockout mice were used to study the Cisd2-dependent effects of hesperetin on skin aging.

**Results:**

Four findings are pinpointed. **Firstly**, in human skin, CISD2 is mainly expressed in proliferating keratinocytes from the epidermal basal layer and, furthermore, CISD2 is down-regulated in the sun-exposed epidermis. **Secondly**, in HEK001 human keratinocytes from an older person, hesperetin enhances mitochondrial function and protects against reactive oxygen species-induced oxidative stress via increased CISD2 expression; this enhancement is CISD2-dependent. Additionally, hesperetin alleviates UVB-induced damage and suppresses matrix metalloproteinase-1 expression, the latter being a major indicator of UVB-induced damage in keratinocytes. **Thirdly**, transcriptomic analysis revealed that hesperetin modulates a panel of differentially expressed genes that are associated with mitochondrial function, redox homeostasis, keratinocyte function, and inflammation in order to attenuate senescence. Intriguingly, hesperetin activates two known longevity-associated regulators, namely FOXO3a and FOXM1, in order to suppress the senescence-associated secretory phenotype. **Finally**, in mouse skin, hesperetin enhances CISD2 expression to ameliorate UVB-induced photoaging and this occurs via a mechanism involving CISD2. Most strikingly, late-life treatment with hesperetin started at 21-month old and lasting for 5 months, is able to retard skin aging and rejuvenate naturally aged skin in mice.

**Conclusions:**

Our results reveal that a pharmacological elevation of CISD2 expression at a late-life stage using hesperetin treatment is a feasible approach to effectively mitigating both intrinsic and extrinsic skin aging and that hesperetin could act as a functional food or as a skincare product for fighting skin aging.

**Supplementary Information:**

The online version contains supplementary material available at 10.1186/s12929-024-01005-w.

## Background

Skin is an important organ that acts as a physical barrier, as well as protecting against ultraviolet (UV) radiation, dehydration, and pathogen infection. Aging leads to a decrease in the structural integrity of skin due to malfunctioning via dysregulation of cellular homeostasis. Skin aging results in fine wrinkles, tissue atrophy with loss of elasticity, increased dryness accompanied by pruritus, increased susceptibility to dermatological disorders and reduced wound healing after injury [1, 2]. Intriguingly, skin aging may also propagate age-related phenotypes to other tissues and organs thereby contributing to systemic whole-body aging via the senescence-associated secretory phenotype (SASP) [3]. Therefore, developing a potent regimen to slow down skin aging and/or to rejuvenate aged skin is of great importance for fighting aging in general.

Both intrinsic and extrinsic factors contribute to skin aging. Intrinsic skin aging is associated with endogenous factors that naturally change with chronological age; these include cellular senescence, mitochondrial dysfunction, decreased antioxidant ability, and perturbations in metabolic homeostasis [1, 3]. Extrinsic skin aging is induced by environmental factors such as UV light. Chronic exposure to UV from sunlight is the main cause of photoaging in sun-exposed skin sites, such as skin on the face and neck [1, 4]. Keratinocytes are the primary type of cells found in the epidermis, the outermost layer of the skin. In humans, keratinocytes constitute about 90% of epidermal cells and play a crucial role in skin aging [4, 5]. Interestingly, senescent keratinocytes are able to produce SASP factors; these include cytokines, extracellular matrix-remodeling enzymes and various other molecules that exert long-range effects on other cells thereby exacerbating the process of aging [6]. In addition, during skin aging, increased mitochondrial damage and elevated levels of reactive oxygen species (ROS) can be observed in keratinocytes [7, 8]. Thus, maintaining mitochondrial integrity and reducing oxidative stress are potential strategies that ought to slow down skin aging.

Many naturally derived bioactive compounds have been shown to delay skin aging and to have a photo-protective capability via their ability to protect mitochondria and via their anti-oxidative properties [9, 10]. Notably, previous studies have revealed that hesperetin, a flavanone aglycone found in citrus fruit peel, exerts several beneficial properties; these include acting as an anti-oxidant agent and as anti-inflammation agent, as well as providing cell protection. All of the above promote skin health [11]. In human dermal fibroblast cells, hesperetin alleviates UVA-induced damage, including suppressing cell death, reducing oxidative stress, and decreasing the expression of matrix metalloproteinases (MMPs) and pro-inflammatory cytokines [12]. In rats, topical treatment with hesperetin-based hydrogels protects against UV-induced skin damage [13]. These studies suggest that hesperetin is a potential anti-aging regimen for skin [14]. However, the molecular mechanism underlying the anti-aging effects of hesperetin is incompletely understood. Additionally, the effects of hesperetin on the senescence of epidermal keratinocytes remained unexplored.

CISD2, a pro-longevity gene, mediates lifespan in mammals. CISD2 is primarily localized within the mitochondrial outer membrane, the endoplasmic reticulum (ER) and the mitochondrial associated ER membranes. Importantly, CISD2 is essential to maintaining mitochondrial function and to regulating intracellular Ca^2+^ homeostasis [15]. Intriguingly, an age-dependent decline of CISD2 expression has been observed in a variety of tissues, including brain, spinal cord, heart, muscle and liver, during the natural aging of mice [16–21]. Additionally, down-regulation of CISD2 expression has been reported in several mouse models of human diseases, namely after spinal cord injury and after cerebral ischemia/reperfusion damage, as well as when human corneal epithelial disorders are present [22–24]. Remarkably, in Cisd2 knockout (Cisd2KO) mice, a premature aging model, the Cisd2KO skin exhibits phenotypes that include a hyperplastic epidermis, an expanded surface, hair follicle atrophy, a decrease in subcutaneous fat and muscle, and an increased thickness of the dermis layer [25]. Conversely, in Cisd2 transgenic (Cisd2TG) mice, a long-lived model, a persistently high level of Cisd2 promotes a healthy lifespan and ameliorates age-related skin degeneration and functional decline. Notably, the age-dependent atrophy of the sebaceous glands, which are the lipid-producing structures associated with the hair follicles, is significantly delayed in Cisd2TG mice. Additionally, the proportion of individual hair follicles associated with sebaceous glands has been shown to significantly increase in old Cisd2TG mice [17]. Interestingly, a study by another group has also shown that CISD2 deficiency results in morphological alterations to mitochondria in the epidermal tissues of Cisd2KO mice, which suggests that CISD2 is involved in the preservation of mitochondrial integrity in keratinocytes [26]. Together, these mouse genetic studies have revealed that maintaining a high level of CISD2 during aging is able to sustain Ca^2+^ homeostasis, balance redox, and preserve mitochondrial function, thereby delaying skin aging.

The beneficial effects of CISD2 whereby there is a slowdown of skin aging in Cisd2TG mice have prompted us to translate the genetic evidence into a pharmaceutical application that is able to enhance CISD2 expression during old age. Specifically, we have identified hesperetin as a potent CISD2 activator that is able to significantly enhance CISD2 expression in the cardiac and skeletal muscles of old mice, thus promoting healthy longevity, while having no detectable in vivo toxicity after long-term treatment (6–7 months) [27, 28]. Here we study the biological effects of hesperetin treatment on aging skin using, firstly, a cell platform, namely an HEK001 human keratinocyte cell line established from an older person, and, secondly, using a mouse model, namely old mice at 21-month old. Specifically, we investigated the anti-aging efficacy of hesperetin on UVB-induced photoaging and naturally aged skin, as well as pinpointing the underlying mechanisms and the potential biological pathways involved.

## Methods

### The human skin tissue array and fluorescent immunohistochemistry (IHC) staining

The human normal skin paraffin tissue microarray (TMA) (SKN1001) used in the present study was purchased from US Biomax Inc. (TissueArray.Com LLC; https://www.tissuearray.com/). Information about SKN1001 can be found on the relevant website (https://www.tissuearray.com/tissue-arrays/Skin/SKN1001). For fluorescent IHC staining, the TMA slides were deparaffinized for 1 h at 65 °C, rehydrated and then antigen-retrieved using target retrieval solution (Dako Denmark A/S, Glostrup, Denmark, S1699). Next, the TMA slides were immersed in 3% H_2_O_2_ in phosphate-buffered saline (PBS) for 15 min at room temperature, followed by blocking with 5% bovine serum albumin (Sigma-Aldrich, Munich, Germany, A7906) in PBS for 1 h at room temperature. After incubation with the various different primary antibodies in antibody diluent (Abcam, Cambridge, England, ab64211) for 16 h at 4 °C, the TMA slides were washed with PBS four times. After washing, the TMA slides were incubated with the appropriate fluorescent secondary antibodies and counter stained with DAPI. Fluorescence images of the TMA slides were captured using a confocal microscope (LSM 900, Zeiss). The following antibodies were used for the fluorescent IHC staining: Cisd2 [29], Cytokeratin 14 (KRT14) (Abcam, ab7800), anti-Rabbit IgG Alexa Fluor™ 647 (Invitrogen, San Diego, CA, USA, A-31573) and anti-Mouse IgG Alexa Fluor™ 488 (Invitrogen, A-11001). The intensity of fluorescence was quantified from the captured images using an illustration and graphic software program (Image J, v1.54; https://imagej.nih.gov/ij/download.html).

### Cell culture and treatment

The HEK001 (CRL-2404, ATCC) human keratinocyte cell line, which was obtained from an older human subject, was cultured in keratinocyte-serum free medium (Gibco, Carlsbad, CA, USA, 17005-042) supplemented with human recombinant EGF (5 ng/mL), gentamicin (10 mg/mL), and 2 mM L-glutamine. The Ker-CT (CRL-4048, ATCC) human neonatal keratinocyte cell line was cultured in KGM Gold BulletKit medium (Lonza, Walkersville, MD, USA, 00192060). These cell lines were maintained in a humidified incubator at 37 °C with 5% CO_2_. The protocols for each specific treatment are described in the figure legends.

### Lentivirus-mediated CISD2 knockdown (KD) in HEK001 keratinocytes

The CISD2 shRNA (Clone ID: TRCN0000282214; 5ʹ-TCCGAAAGTAGTGAATGAAAT-3ʹ) and control luciferase shRNA (Clone ID: TRCN0000231719, shLuc; 5ʹ-GCGGTTGCCAAGAGGTTCCAT-3ʹ) were separately packaged in lentivirus and were generated by the National RNAi Core Facility at Academia Sinica in Taiwan. The HEK001 keratinocytes were infected with each lentivirus individually and subjected to selection for 72 h in growth medium containing puromycin (3 μg/mL) (Invitrogen, A11138-03). The two stable lines thus obtained were then maintained in complete growth medium containing puromycin (1.5 μg/mL). The KD efficiency of CISD2 was examined by Western blot analysis.

### Analysis of pro-MMP-1 levels in the conditioned medium

Pro-MMP-1 levels in the conditioned medium obtained from HEK001 cells were analyzed using a human Pro-MMP-1 ELISA kit (R&D Systems, Minneapolis, MN, USA, DMP100) according to the manufacturer’s instructions.

### RNA isolation and real-time reverse transcription qPCR

Total RNA was isolated from cells using TRI Reagent (Sigma-Aldrich, T9424). Reverse transcription and real-time quantitative PCR were performed as previously described [18]. The cDNA was synthesized by reverse transcription of total RNA using random hexamers (Roche, Basel, Switzerland, 11034731001) and SuperScript™ III reverse transcriptase (Invitrogen, 18080) according to the manufacturer’s instructions.

### Measurement of mitochondrial oxygen consumption rate by HEK001 keratinocytes

Mitochondrial oxygen consumption rate (OCR) was measured using a XFe24 analyzer (Seahorse Bioscience, North Billerica, MA, USA) as previously described [29]. Briefly, HEK001 cells were cultured on a XF24 V7 plate to give 7 × 10^3^ cells/well after the HEK001 cells had been kept in normal growth medium for 24 h. The medium was then replaced by hesperetin containing growth medium for 48 h. After hesperetin treatment for 48 h, the culture medium was replaced by fresh normal growth medium for 1 h before OCR measurement. The OCR was measured at 37 °C before and after adding the indicated chemicals (1 μM oligomycin A [an ATP synthase inhibitor] and 0.5 μM rotenone [a mitochondrial complex I inhibitor] with 0.5 μM antimycin A [a mitochondrial complex III inhibitor]). This was in order to monitor the OCR contributed by mitochondrial basal respiration and ATP-linked respiration, and by non-mitochondrial respiration [30]. The results are presented in pmol/minute/μg protein.

### Mitochondria membrane potential assay

For the mitochondria membrane potential assay, HEK001 cells were stained using 2 μM JC-1 dye (Thermo Fischer Scientific, Waltham, MA, USA, T3168) in culture medium for 30 min at 37 °C in the dark. After this, the ratio of red to green was measured by confocal microscopy (LSM700, ZEISS, Jena, Germany) before and after H_2_O_2_ (98 μM) treatment.

### Mouse models and hesperetin treatment

Cisd2KO mice were generated as previously described [25]. All mice used in this study are males with pure or congenic C57BL/6 backgrounds. All mice were bred and housed in a specific pathogen-free facility at a constant room temperature (20–22 °C) with a 12 h light and 12 h dark cycle (7 a.m–7 p.m.). For the anti-aging study, the mice were fed ad libitum with AIN-93G (TestDiet, St. Louis, MO, USA) diet mixed with hesperetin (0.07% [w/w]; Sigma-Aldrich, H4125; 100 mg/kg/day) or mixed with vehicle (3.04% propylene glycol [w/w]; Sigma-Aldrich, 16033). To evaluate the protective effect of hesperetin on UVB-induced skin damage, the mice were treated with hesperetin (30 mg/kg/day) or vehicle by a feeding tube. For the UVB treatment, the UVB apparatus consisted of four UVB lamps (G4T5E, SANKYO DENKI, Hiratsuka, Kanagawa, Japan); the spectral wavelength range of the UVB lamps was 280–360 nm, and peak light source intensity was 306 nm. Mice under anesthesia were placed individually in a plastic box with UVB lamps; the fluence of UVB on the mouse dorsal surface was 349 mJ/cm^2^ for 75 s [31, 32]. Mice were treated with hesperetin or vehicle for 7 days before UVB irradiation followed by UVB irradiation for 5 consecutive days as hesperetin or vehicle treatment continued. The dorsal skins were dissected two days after the final UVB treatment. After each specific treatment, the mice were sacrificed by CO_2_ inhalation, which is a humane method of euthanasia. The animal protocol was approved by the Institutional Animal Care and Use Committee (No. 1040103) of National Yang Ming Chiao Tung University. The animal protocols were designed to follow the associated guidelines and the 3R principles (Replacement, Reduction and Refinement) in accordance with the “Animal Protection Act” of Taiwan.

### Histopathology

Tissue sections of the dorsal skin were collected, fixed with 10% neutral buffered formalin, and embedded in paraffin. Tissue section (10 μm) were subjected to Masson’s trichome staining using a standard procedure as previously described [17]. The thicknesses of the dermis and epidermis were quantified by random measurements of the thickness of these regions in each skin sample using SPOT Imaging Software Advance (Diagnostic Instruments, Inc., Sterling Heights, MI, USA). The hair follicle density, the sebaceous gland per hair follicle density and the nuclei per sebaceous gland density were quantified by random measurements of individual skin samples.

### Western blot analysis

Skin tissue and cell samples were homogenized in RIPA lysis buffer (50 mM Tris at pH 7.4, 150 mM NaCl, 0.5% Sodium deoxycholate, 0.1% SDS and 1% Triton X-100 with complete protease inhibitor and phosphatase inhibitor cocktails [Roche]) and then denatured in SDS sample buffer (50 mM Tris at pH 6.8, 100 mM dithiothreitol, 2% SDS and 10% glycerol) for 10 min at 100 °C. The extracted proteins were separated by SDS–polyacrylamide gel electrophoresis (Bio-Rad, Hercules, CA, USA), followed by electro-transferred to a polyvinylidene fluoride membrane (PerkinElmer, Waltham, MA, USA). The membranes were blocked with 5% (w/v) non-fat dried milk solution, then incubated with the required specific antibody. This was followed by detection by visualizer kit (Millipore, Burlington, MA, USA, WBKLS0500). The following antibodies were used for the Western blotting: Cisd2 [29], Gapdh (Millipore, MAB374), β-tubulin (Millipore, 05661) and MMP-1 (Proteintech, Rosemont, IL, USA, 10371-2-AP).

### Cell and tissue ROS level analysis

Intracellular ROS levels in HEK001 cells were determined using a ROS sensitive fluorescence dye, DCF-DA (Molecular Probes). The ROS and reactive nitrogen species (RNS) levels were detected in the skin tissue samples using a ROS/RNS Assay Kit (Cell Biolabs, San Diego, CA, USA, STA-347) according to the manufacturer’s instructions.

### RNA-seq analysis

The RNA sequencing and pathway analyses were conducted as previously described [20, 27]. Briefly, the RNA sequencing was performed by National Yang Ming Chiao Tung University Genomics Center for Clinical and Biotechnological Applications. The dataset was generated to a depth of at least 20 million reads for each sample by single-end sequencing. After the mapping, the unique gene reads were analyzed as expected counts to assess gene expression. A total of 12,103 genes were analyzed after being filtered to identify the genes expressed in the HEK001 keratinocytes (minimal expected counts > 50 detected in at least 50% samples). The normalized counts and differentially expressed genes (DEGs) were obtained using DESeq2 (using the Wald test) and the overall false discovery rate (FDR) was controlled to be below 0.005 with an absolute fold change > 1.5. The enrichment for Gene Ontology (GO) annotation and for KEGG pathway analysis was conducted using the online tool STRING (https://string-db.org). The canonical pathway analysis and upstream analysis were conducted using QIAGEN Ingenuity Pathway Analysis (IPA) software (Ingenuity Systems^®^, www.ingenuity.com). The values of the normalized counts were transformed into z-scores (normalized counts minus mean and divided by standard deviation [SD]) and these scores were used to create heatmaps using Multi Experiment Viewer 4.9 software (mev.tm4.org).

### Statistical analysis

The data are presented as mean ± SD as described in the figure legends. Comparisons between two groups were carried out using unpaired two-tailed Student’s t tests. Comparisons among more than two groups were carried out using one-way ANOVA with the Bonferroni multiple comparison test. When statistical differences among groups were analyzed, *p* < 0.05 was considered to be significant. Statistical analysis was conducted using GraphPad Prism software (v9.0, GraphPad Software, San Diego, CA, USA).

## Results

### CISD2 is mainly expressed in the proliferating keratinocytes of epidermal basal layer and is down-regulated in sun-exposed sites of the human skin epidermis

**Fig. 1:**
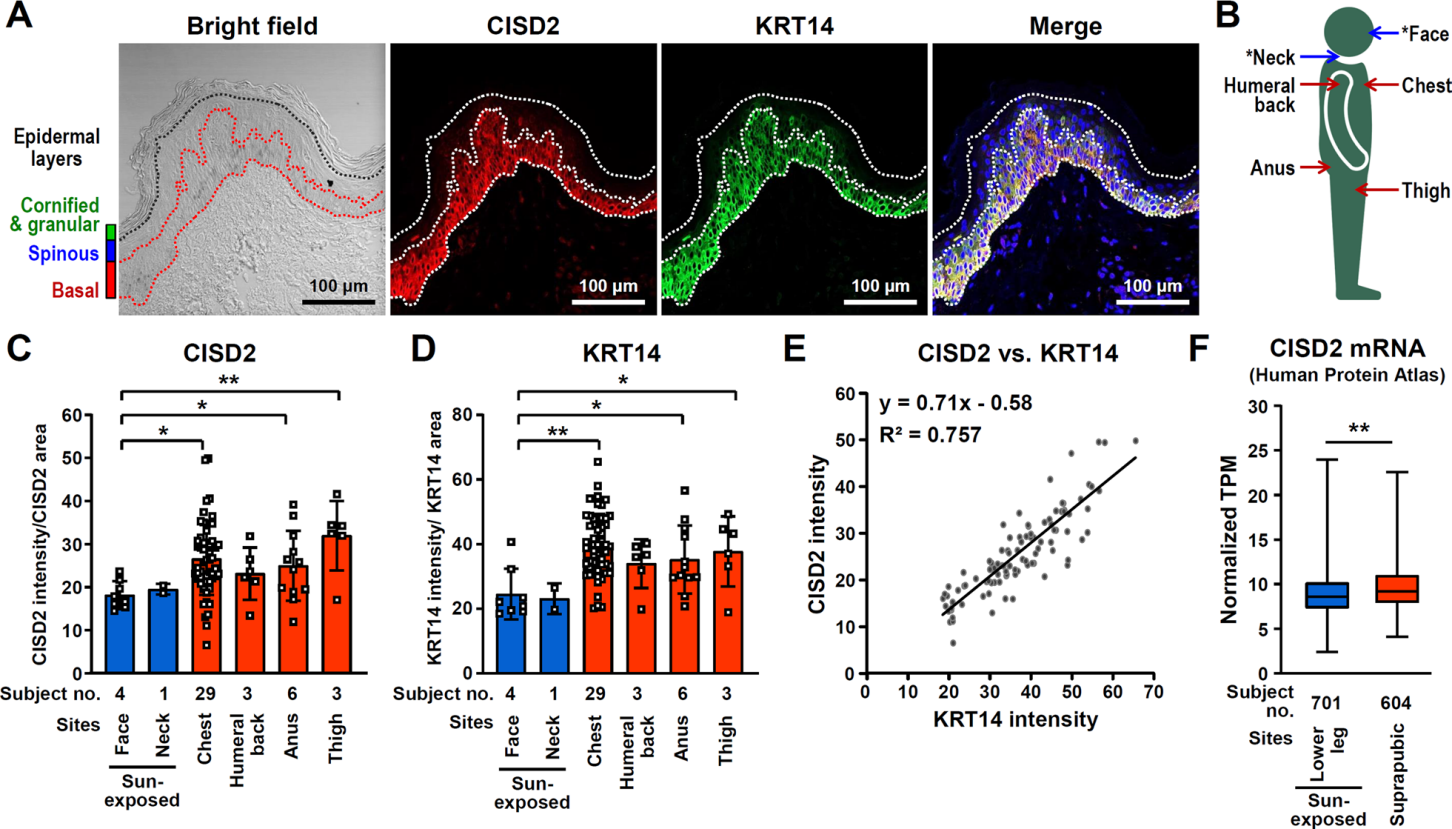
CISD2 expression is down-regulated in sun-exposed sites of the human skin epidermis.** A** Fluorescent immunohistochemistry (IHC) staining of CISD2 and KRT14 (a marker of the proliferating keratinocytes in the epidermis) in normal human skin. **B** The icon shows the various collection sites on normal human skin that are present in the human skin tissue array (SKN1001). The sun-exposed sites are marked with an asterisk. **C** Quantification of CISD2 intensity in the various different sites of human normal skin. **D** Quantification of KRT14 intensity in various different sites of human normal skin. **E** Correlation analysis between CISD2 and KRT14 intensity. **F** CISD2 mRNA expression in the human skin from the suprapubic area (non-sun-exposed) and the lower leg area (sun-exposed). These data were collected from the Human Protein Atlas database (https://www.proteinatlas.org). Data are presented as mean ± SD. *p < 0.05; **p < 0.005. The statistical analysis was performed by Student’s t test

The composition of cell types and the anatomical structure of the skin varies across different body areas. These differences are primarily driven by unique functional requirements and the environmental exposures that each region of the body experiences [33]. To analyze the expression pattern of CISD2 in human skin, we obtain a skin tissue array (SKN1001) that contains normal skin samples collected from various body sites on human subjects and perform immunofluorescent staining to detect the presence of CISD2 and KRT14 (a marker for basal layer keratinocytes). Interestingly, we found that CISD2 is mainly expressed in the proliferating keratinocytes present in the epidermal basal layer and that CISD2 protein expression is dramatically lower in spinous layer keratinocytes and disappears in the granular layer keratinocytes (Fig. 1A and Additional file 1: Fig. S1A-D). Moreover, the expression level of CISD2 protein was found to be significantly decreased in sun-exposed skin, namely the face and neck, compared to skin tissue samples obtained from the chest, humeral back, anus, and thigh, which are areas usually protected from sunlight (Fig. 1B–D and Additional file 1: Fig. S1E and S1F). Intriguingly, the expression level of CISD2 protein is positively correlated with the expression level of KRT14 protein in the keratinocytes of human skin (Fig. 1E). Previous studies have revealed that CISD2 deficiency impairs cell proliferation in corneal epithelial cells and in cancer cell lines [15, 24]. In addition, KRT14 deficiency also reduces cell proliferation and delays cell cycle progression in HaCaT human keratinocytes under KRT14 knockdown [34]. Accordingly, CISD2 is likely to be involved in the proliferation of basal keratinocytes. Furthermore, based on information obtained from the Human Protein Atlas database (https://www.proteinatlas.org) [35], CISD2 is also expressed in epidermal keratinocytes, Langerhans cells and melanocytes, but such expression is not detectable in dermal fibroblasts. Notably, the level of CISD2 mRNA is significantly lower in the skin of the lower leg, which is more accessible to sunlight, compared with the suprapubic skin, which is usually well protected from sunlight (Fig. 1F). These results indicate that CISD2 is mainly expressed in the proliferating keratinocytes of the epidermal basal layer and that sunlight exposure is likely to down-regulate CISD2 expression.

### Hesperetin improves mitochondrial function and decreases oxidative stress via an enhancement of CISD2 expression in HEK001 human keratinocytes obtained from an older person

**Fig. 2:**
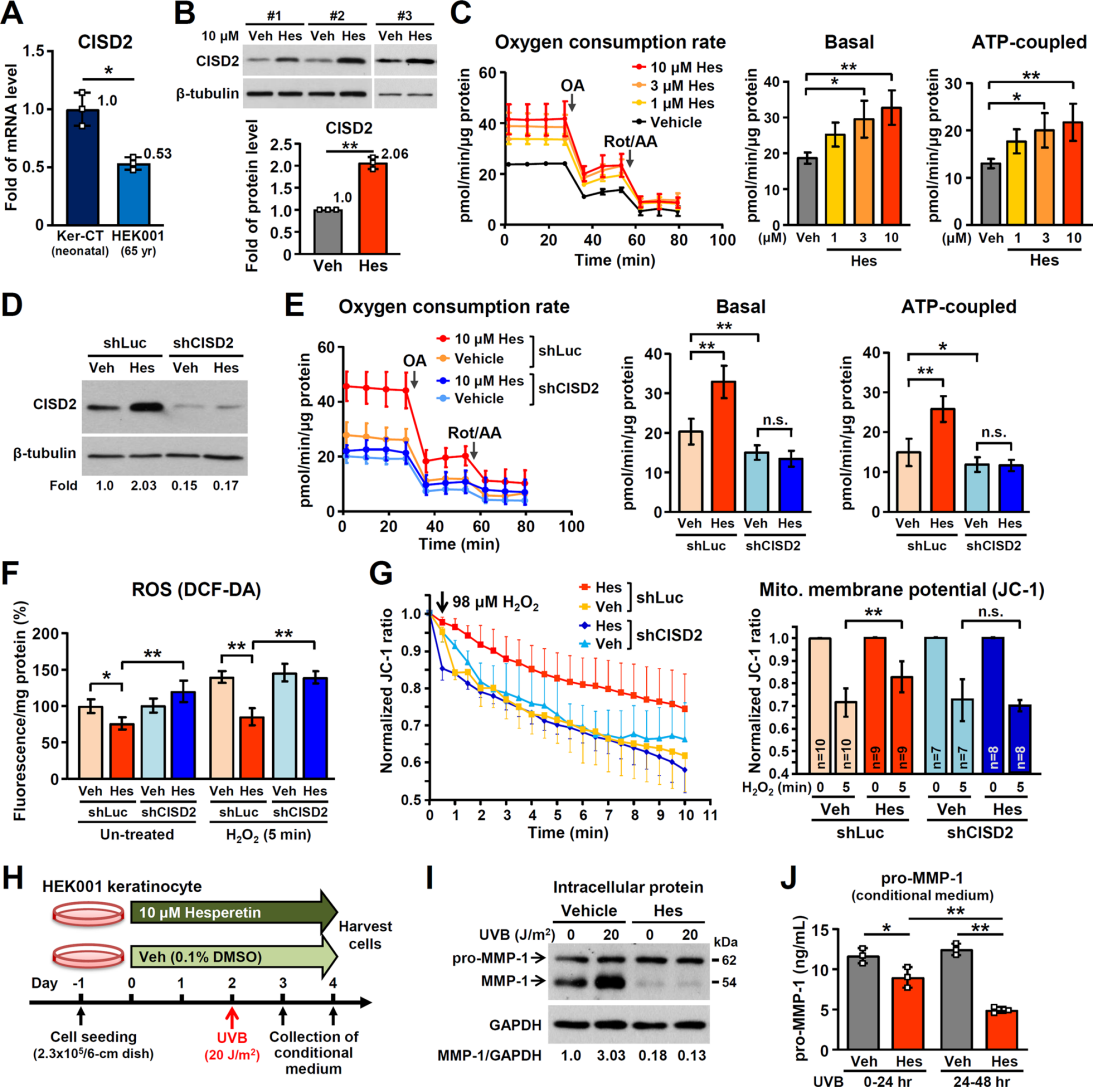
Hesperetin enhances CISD2, promotes mitochondrial function, alleviates oxidative stress and UVB-induced damage in HEK001 keratinocytes. **A** Real-time RT-qPCR of CISD2 mRNA in Ker-CT and HEK001 keratinocytes. CISD2 levels were normalized to HPRT1. **B** Western blot analysis of CISD2 protein in the vehicle (Veh)- and hesperetin (Hes)-treated HEK001 keratinocytes. **C** The mitochondrial oxygen consumption rates (OCR) of HEK001 keratinocytes after different treatments (n = 10–12). OA, Oligomycin A; Rot/AA, rotenone/antimycin A. **D** Western blot analysis of CISD2 protein in the shLuc and CISD2 knockdown (KD) HEK001 keratinocytes. **E** The mitochondrial OCR of HEK001 keratinocytes after different treatments (n = 8). **F** Intracellular H_2_O_2_ levels. To assess H_2_O_2_-induced oxidative stress, HEK001 keratinocytes were treated with 5 μM H_2_O_2_ for 5 min before monitoring the intracellular H_2_O_2_ levels. **G** Mitochondrial membrane potential and quantification of the red/green ratios by JC-1 staining of HEK001 keratinocytes. For hesperetin treatment, 10 μM hesperetin was used to treat the shLuc and CISD2 KD HEK001 keratinocytes for 48 h and mitochondrial membrane potential was monitored before and after 98 μM H_2_O_2_ treatment. **H** Protocol for the treatment with hesperetin and its effect on UVB-induced MMP-1 activation in HEK001 keratinocytes. **I** Western blot analysis of MMP-1 protein in the Veh- and Hes-treated HEK001 keratinocytes after UVB exposure. **J** ELISA analysis of pro-MMP-1 protein in the conditioned medium from various groups of HEK001 keratinocytes. Vehicle, 0.1% DMSO. All the experiments were performed and repeated three independent times as biological replicates using HEK001 keratinocytes. Data are presented as mean ± SD. In (**C** and **J**), the statistical analysis was performed by one-way ANOVA with Bonferroni multiple comparison test. In (**E** and **F**), the statistical analysis was performed by two-way ANOVA with Bonferroni multiple comparison test. In (**A**), (**B**) and (**G**), the statistical analysis was performed using Student’s t test; not significant (n.s.)

It should be noted that the expression level of CISD2 mRNA is significantly lower in old keratinocytes that form the HEK001 cell line, which was established from the skin of a 65-year-old male, compared with keratinocyte from the neonatal Ker-CT cell line, which was established from a neonatal human foreskin. This suggests that CISD2 is down-regulated during the keratinocyte senescence process (Fig. 2A). Previously we have identified hesperetin as a potent Cisd2 activator [27]. Remarkably, when HEK001 keratinocytes are treated with hesperetin (10 μM), there is a significant increase in CISD2 expression level by about twofold after treatment for 48 h (Fig. 2B). Cell proliferation analyses using different concentrations of hesperetin (1, 3, 10, 30 and 100 μM) showed no overt effect on cell proliferation; in addition, there was no cytotoxic effect at a concentration lower than 30 μM (Additional file 1: Fig. S2A & S2B).

To study if hesperetin is able to enhance mitochondrial function in HEK001 keratinocytes, we measured mitochondrial OCR after hesperetin treatment. Notably, hesperetin increases mitochondrial OCR, including basal and ATP-coupled respiration (Fig. 2C). Furthermore, in order to investigate if hesperetin functions in a CISD2-dependent manner, we carry out CISD2 KD (shCISD2) before the treatment (Fig. 2D). Intriguingly, both the basal and ATP-coupled respiration are significantly decreased in HEK001-shCISD2 cells compared with HEK001-shLuc control cells treated with vehicle. Importantly, hesperetin loses its beneficial effect with respect to the enhancement of mitochondrial OCR in HEK001-shCISD2 cells (Fig. 2E). This result suggests that CISD2 plays a crucial role in mitochondrial function and that the beneficial effects of hesperetin are dependent on a mechanism that involves CISD2 expression in HEK001 keratinocytes.

To study if hesperetin exerts its antioxidant ability via an upregulation of CISD2, we measured ROS levels in HEK001-shCISD2 and HEK001-shLuc cells that were undergoing H_2_O_2_ challenge. We found that hesperetin is able to decrease the basal level of ROS in the absence of the H_2_O_2_ challenge, and this also occurred under the H_2_O_2_ challenge. However, these antioxidant effects disappeared in HEK001-shCISD2 cells (Fig. 2F). To study if upregulation of CISD2 by hesperetin can protect the HEK001 keratinocytes against ROS-induced mitochondrial dysfunction, we measure the time course of changes in mitochondrial membrane potential by JC-1 staining after H_2_O_2_ (98 μM) challenge. Under these conditions, a decrease in the red/green fluorescence ratio indicates a depolarization of mitochondrial membrane potential. Our result revealed that hesperetin significantly slows down the H_2_O_2_-induced decline in mitochondrial membrane potential of HEK001-shLuc cells. However, no overt antioxidant effect of hesperetin was observed in HEK001-shCISD2 cells (Fig. 2G and Additional file 1: Fig. S2C). Taken together, these results demonstrate that hesperetin enhances mitochondrial function and protects against oxidative stress in HEK001 human keratinocytes via an upregulation of CISD2 expression and that it functions primarily in a CISD2-dependent manner.

### Hesperetin alleviates UVB-induced cellular damage in human keratinocytes from an elderly male.

UV radiation is one of the major environmental factors that trigger skin aging. Excessive UVB exposure leads to damage keratinocytes in the epidermis [36]. To examine the protective effect of hesperetin on UVB-induced damage in keratinocytes from an elderly human source, we pre-treated HEK001 with hesperetin for 2 days before exposing the cells to UVB radiation. The cells were treated with one dose of UVB radiation (20 J/m^2^) and then cultured for another 2 days before harvesting. The conditioned medium was collected at 24 h and 48 h after UVB radiation treatment (Fig. 2H). Previous studies have shown that aberrant upregulation of matrix metalloproteinase-1 (MMP-1), which is a secretory interstitial collagenase that mediates collagen degradation and extracellular matrix remodeling, contributes to skin aging. Moreover, elevation of MMP-1 is able to serve as a biomarker of UVB-induced damage in keratinocytes [6, 37–39]. Indeed, Western blot analysis of the various HEK001 keratinocyte samples revealed that intracellular MMP-1 expression is dramatically increased after UVB exposure. Strikingly, hesperetin down-regulates the levels of intracellular MMP-1 in HEK001 keratinocytes under both the basal conditions with no radiation and after UVB radiation (Fig. 2I). In addition, hesperetin treatment also decreases the levels of pro-MMP-1 detected by immunoassay in the conditioned medium (Fig. 2J). These results indicate that hesperetin is able to alleviate UVB-induced damage in HEK001 keratinocytes.

### Hesperetin improves UVB-induced skin photoaging in mice via an increase in CISD2 expression

**Fig. 3:**
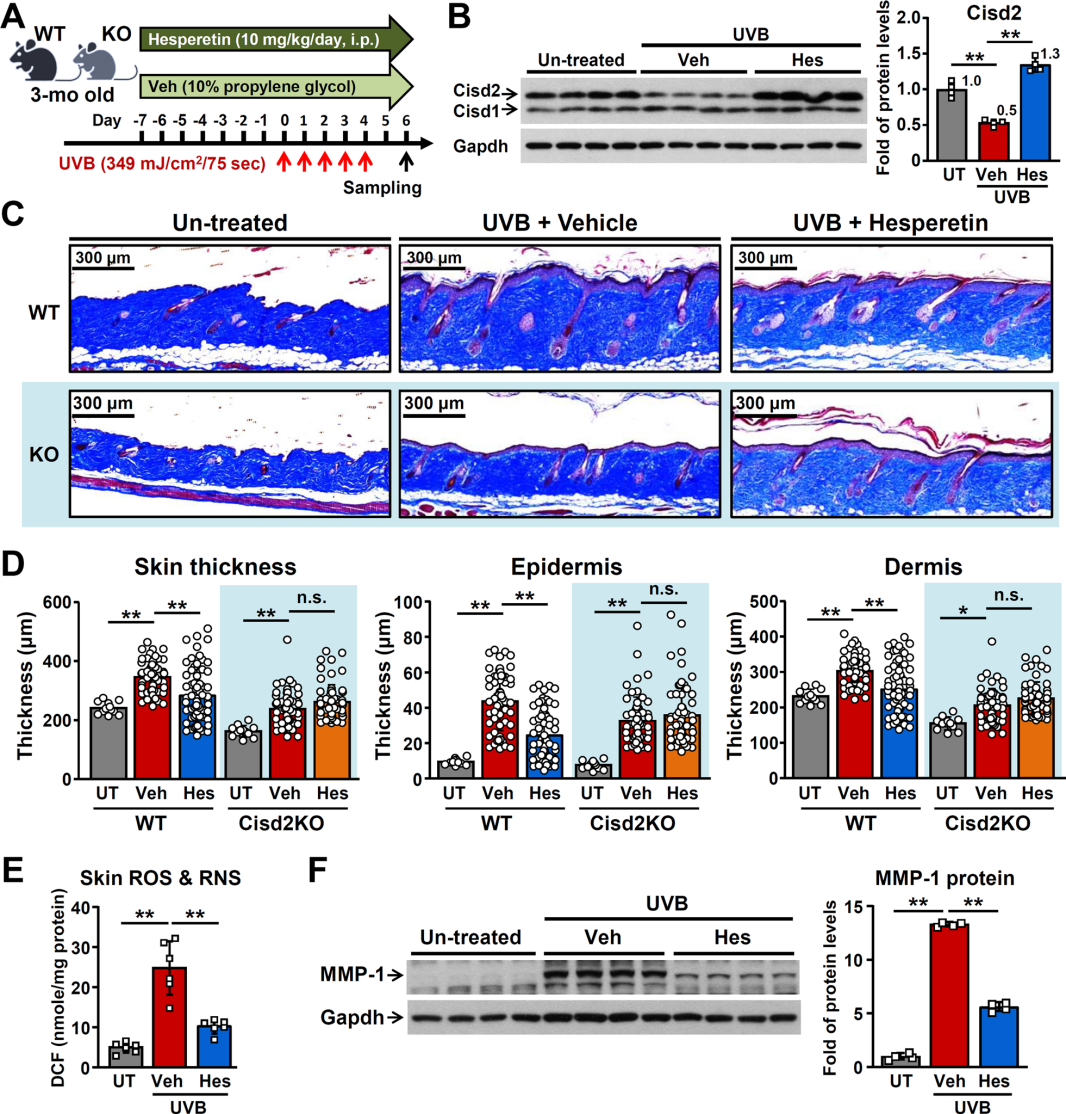
Hesperetin ameliorates UVB-induced skin photoaging in a Cisd2-dependent manner. **A** The protocol for treatment with hesperetin and its effect on UVB-induced skin damage in WT and Cisd2 KO mice at 3-month old. The mice were pre-treated with hesperetin (10 mg/kg/day, i.p. injection) for 7 days, and then exposed to UVB (312 nm, 349 mJ/cm^2^/75 s) light once a day for 5 days in a UVB box. Mice were sacrificed at 6 days after the first UVB exposure. **B** Western blot analysis of the level of Cisd2 protein in the skin of Veh-treated or Hes-treated mice after UVB exposure. **C** Masson’s trichrome staining of skin sections from the various different groups of mice. UVB exposure significantly induced skin damage and increases skin thickness, which is one of the major characteristics of photoaging. **D** Quantification of the total, epidermal and dermal layers of skin thickness in various different groups of mice. Hesperetin treatment ameliorates UVB-induced skin photoaging in the WT mice. However, hesperetin has no obvious beneficial efficacy when Cisd2 is absent in the Cisd2 KO mice. **E** Total reactive oxygen and nitrogen species (ROS & RNS) levels in the skin tissues of Veh-treated or Hes-treated WT mice after UVB exposure. **F** Western blot analysis of MMP-1 protein (a marker of UV damage) in the skin of Veh-treated or Hes-treated WT mice after UVB exposure. Data are presented as mean ± SD. *p < 0.05; **p < 0.005 by one-way ANOVA with Bonferroni multiple comparison test. *UT* untreated

To evaluate the effect of hesperetin on UVB-induced skin photoaging, we pre-treated mice with hesperetin (10 mg/kg/day, i.p. injection) for 7 days and then exposed them to UVB; skin tissue samples were collected 2 days after the end of radiation (Fig. 3A). Notably, the level of CISD2 protein was found to be significantly decreased by 50% in the skin samples after UVB damage. Remarkably, hesperetin treatment not only attenuated the UVB-induced CISD2 down-regulation but also dramatically increased the level of CISD2 protein by about 2.6-fold compared with the vehicle control skin samples (Fig. 3B). After UVB irradiation, the dorsal skin samples of vehicle-treated wild-type (WT) mice exhibited significant redness and had thick wrinkles on their skin surface. By way of contrast, hesperetin protected the skin from these gross phenotypic changes, as shown by the presence of smooth and unwrinkled skin on the hesperetin-treated mice (Additional file 1: Fig. S3A). Furthermore, histopathological analysis also revealed that UVB irradiation resulted in a significant increase in the thickness of the skin, including both the epidermis and dermis layers. In contrast, hesperetin treatment significantly decreased the thickening of skin that occurs after UVB damage (Fig. 3C and D). Moreover, UVB treatment resulted in a significant increase in ROS and RNS levels, as well as the level of intracellular MMP-1 protein, in the skin samples from vehicle-treated WT mice. Hesperetin, on the other hand, significantly attenuated these indicators of UVB-induced damage to the skin (Fig. 3E and F).

To delineate if the beneficial effects of hesperetin in relation to UVB-induced damage function in a CISD2-dependent manner, we carry out the UVB radiation protocol on Cisd2KO mice (Fig. 3A). Notably, in Cisd2KO mice, hesperetin lost its beneficial effects with respect to protecting from UVB-induced skin damage in terms of the gross view of the dorsal skin, when assessed by the redness remaining on the surface of their skin after hesperetin treatment (Additional file 1: Fig. S3A), and in terms of the thickness of the skin and the thickness of epidermis and dermis layers (Fig. 3C and D). We next evaluated the effectiveness of various different administration routes. We performed the protocol using oral administration of hesperetin (Additional file 1: Fig. S3B) and our results revealed that oral administration is just as effective as the intraperitoneal administration of hesperetin in terms of UVB-induced damage protection of the skin (Additional file 1: Fig. S3C-E). Together, these results reveal that hesperetin is able to ameliorate UVB-induced skin damage and show that hesperetin functions mainly in a manner that is dependent on CISD2 expression. Specifically, hesperetin loses its beneficial effects in the Cisd2KO mice where we know that CISD2 is absent.

### Hesperetin rejuvenates naturally aged skin in old mice

Interestingly, topical treatment with hesperetin (10 μM) is able to enhance CISD2 reporter expression in ear skin from a CISD2 reporter mice [40] (Additional file 1: Fig. S4A). Furthermore, CISD2 protein level is significantly decreased in the skin of middle-aged mice (14-month old) and old mice (24-month old) compared to mice at a young age (3-month old) (Additional file 1: Fig. S4B). This is consistent with our previous findings that there is an age-dependent decline in CISD2 expression in a variety of tissues during aging. Furthermore, hesperetin treatment (10 mg/kg/day, i.p. for 30 days) significantly increases the CISD2 protein level in the skin of middle-aged WT mice (4-month old) (Additional file 1: Fig. S4C).

**Fig. 4:**
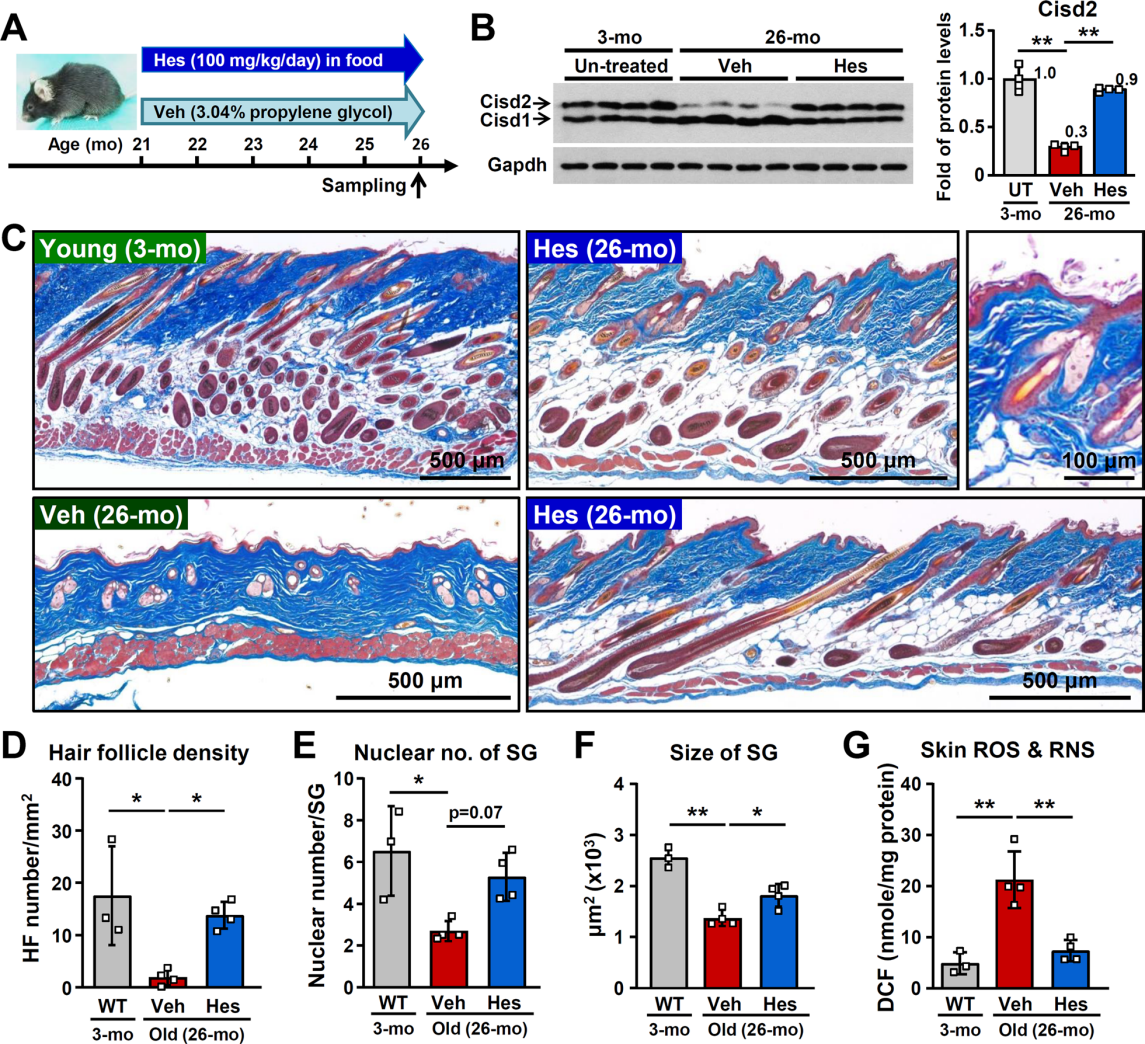
Hesperetin rejuvenates the naturally aged skin of WT mice. **A** For the hesperetin (Hes) treatment, 21-month old aged WT male mice were fed dietary hesperetin (100 mg/kg/day provided in food) or vehicle (Veh) control food (3.04% propylene glycol, w/w) for 5 months and then sacrificed at 26-months old. **B** Western blot analysis and quantification of Cisd2 protein levels in the skin of 26-months old mice treated with dietary hesperetin or vehicle control food for 5 months (starting at 21-months old). The protein level of Cisd2 in 3-months old WT mice serves as a young mouse control. **C** Masson’s trichrome staining of skin from the various different groups of mice. **D** Quantification of hair follicle density. The numbers of hair follicles present were normalized against the length measured. **E** Quantification of nuclear number of the sebaceous glands (SGs) in the skin of mice. **F** Quantification of the size of the sebaceous glands in the skin of mice. **G** Total reactive oxygen and nitrogen species (ROS & RNS) levels in skin tissue samples from young mice at 3-months old and Veh-treated or Hes-treated old mice at 26-months old. Data are presented as mean ± SD. *p < 0.05; **p < 0.005 by one-way ANOVA with Bonferroni multiple comparison test. *UT* untreated

To investigate if hesperetin is able to delay or even rejuvenate naturally aged skin, we treated old mice (21-month old) with dietary hesperetin (100 mg/kg/day provided in food) for 5 months and sacrificed them at 26-month old (Fig. 4A). The levels of CISD2 in these mice were found to be significantly decreased in the skin from 26-month old vehicle-treated mice. However, dietary hesperetin was found to increase CISD2 protein levels in skin samples from old mice reaching a level comparable to that of young mice at 3-month old (Fig. 4B). This result shows that the CISD2 level in skin is able to be targeted pharmaceutically by dietary hesperetin at a late stage of life. During natural aging, multiple histopathological phenotypes [33] can be detectable in the skin of vehicle treated old mice at 26-month old; these include decreased skin thickness, reduced subcutaneous fat, hair loss, and sebaceous gland atrophy (Fig. 4C). Strikingly, hesperetin treatment alleviates all of these age-related deleterious changes, this includes increasing hair follicle density (Fig. 4D), increasing the size of sebaceous glands and increasing the nuclear number per sebaceous gland (Fig. 4E and F). Furthermore, an increase in ROS levels is known to be one of the main factors that promote skin aging [7]. Notably, the levels of ROS and RNS in skin were both significantly decreased after hesperetin treatment of old mice (Fig. 4G). Collectively, these results indicate that late-life treatment with dietary hesperetin is capable of enhancing CISD2 expression in the naturally aged skin of old mice. As a result of the enhanced CISD2 expression, age-associated skin phenotypes are much improved and even rejuvenated to some extent in terms of histopathology.

### Hesperetin attenuates cellular senescence and maintains redox homeostasis

**Fig. 5:**
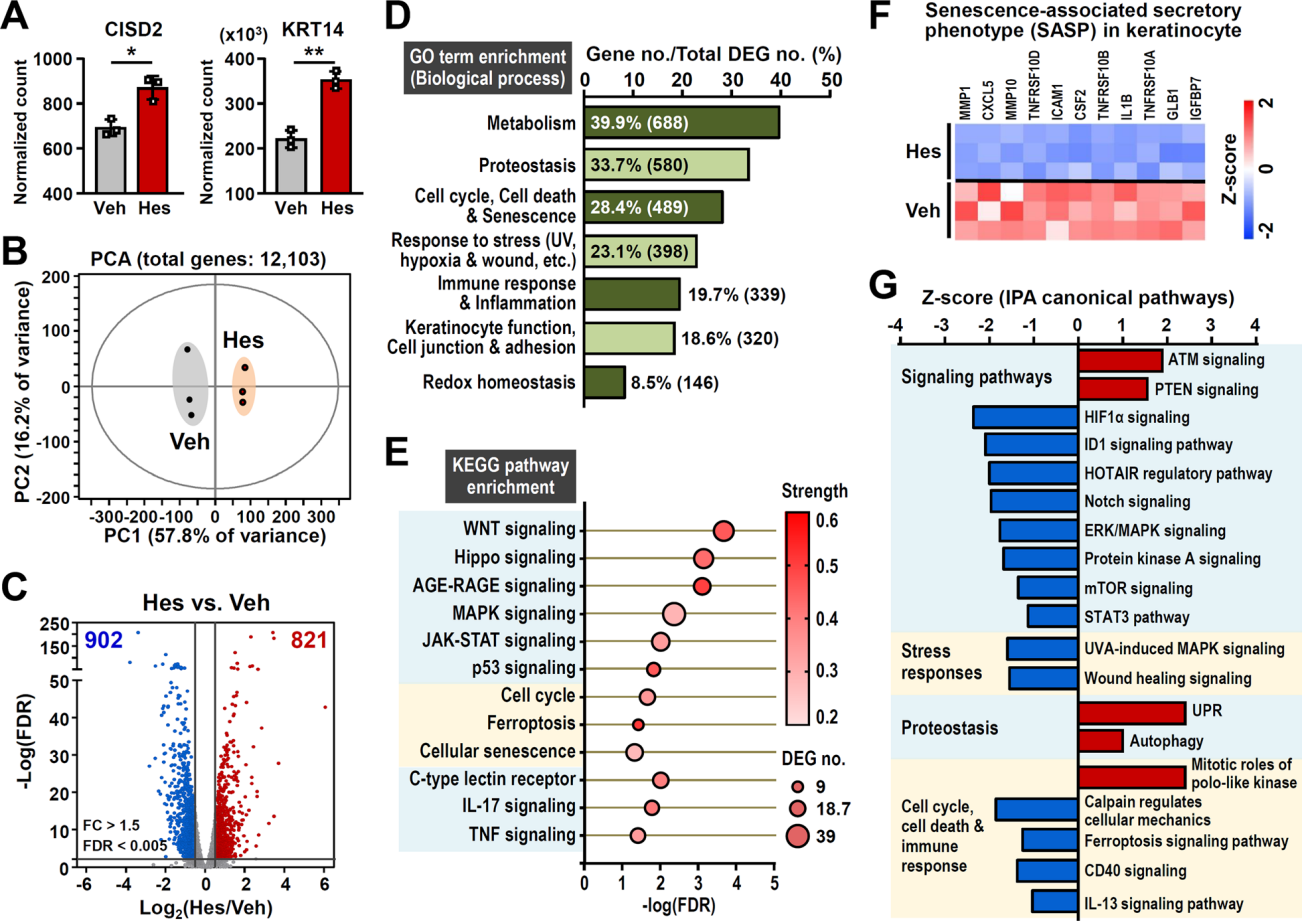
Hesperetin modulates a panel of DEGs to attenuate senescence and maintain cellular homeostasis. **A** CISD2 and KRT4 mRNA levels are up-regulated in the HEK001 keratinocytes after hesperetin (Hes) treatment for 48 h. Vehicle (Veh), 0.1% DMSO. **B** Principal component analysis (PCA, EZinfo 3.0.3 software) of all transcriptomic data (12,103 genes) from vehicle or hesperetin treated HEK001 keratinocytes (n = 3 independent trials). **C** Volcano plot revealing transcriptome change (Hes *vs*. Veh). Horizontal line shows the 0.5% false discovery rate (FDR) threshold. Red or blue plots identify genes above the indicated FDR and fold change threshold. A total of 1723 differentially expressed genes (DEGs) were found to be modulated by hesperetin (821 up-regulated and 902 down-regulated genes; Hes *vs* Veh, Fold change > 1.5 and FDR < 0.005). **D** The biological processes obtain from Gene Ontology (GO) annotation of transcriptome changes (1723 DEGs) after hesperetin treatment. **E** A bubble plot illustrating the KEGG pathway enrichment of the DEGs after hesperetin treatment. The grouping of the GO annotation and KEGG pathways were carried out by STRING v11.5 (https://string-db.org/). Pathway FDR < 0.05. **F** A heatmap illustrating that hesperetin down-regulates the expression of a panel of SASP-related factors (Hes *vs* Veh, Fold change > 1.1 and *p* < 0.05). **G** Canonical pathway analysis by Ingenuity Pathway Analysis (IPA) software based on Hes-mediated transcriptome changes (Hes *vs*. Veh; pathway *p*-value < 0.05). Data are presented as mean ± SD. *p < 0.05; **p < 0.005 by Student’s t test. *DEGs* differentially expressed genes, *FC* fold change, *Redox* reduction–oxidation, *SASP* senescence-associated secretory phenotype, *UPR* unfolded protein response, *UV* ultraviolet

In order to explore the molecular mechanisms underlying the beneficial effects of hesperetin, we performed RNA sequencing (RNA-seq) and pathway analysis on HEK001 human keratinocytes from an elderly human with and without hesperetin treatment for 48 h. The mRNA levels of 12,103 genes were quantified. RNA-seq analysis revealed that hesperetin enhances the level of CISD2 mRNA as well as the level of KRT14 mRNA (Fig. 5A). This is consistent with the findings that, in human skin, there is a positive correlation between CISD2 and KRT14 protein levels in keratinocytes (Fig. 1E). Principal component analysis showed a dramatic difference in the transcriptome profiles between the vehicle-treated and hesperetin-treated keratinocytes (Fig. 5B). Next a pair-wise DEG analysis was performed, specifically, normalized counts and DEGs were obtained using DESeq2 (with the Wald test). During this analysis the overall FDR was controlled to be below 0.005 with an absolute gene expression fold change > 1.5 (Hes vs. Veh, either up-regulated or down-regulated). A total of 1723 DEGs of which 821 were up-regulated and 902 were downregulated under the influence of hesperetin (Fig. 5C).

The DEGs were annotated using GO and KEGG pathway analysis. Functional enrichment analysis of the GO classification (biological processes) revealed that the DEGs are associated with metabolism, proteostasis, cell cycle, cell death and senescence, stress response, inflammation and immune response, keratinocyte functioning and redox homeostasis (Fig. 5D and Additional file 1: Fig. S5A-S5D). In addition, the GO and KEGG pathway enrichment analyses indicate that the DEGs are involved in a number of important signaling pathways, including WNT, Hippo, AGE-RAGE, MAPK, JAK-STAT and p53, as well as the cell cycle, ferroptosis, cellular senescence and inflammation associated signaling (Fig. 5E and Additional file 1: Fig. S5E and S5F). Remarkably, eleven factors involved in the SASP of keratinocytes are significantly down-regulated after hesperetin treatment [41] (Fig. 5F).

IPA of the DEGs identified several significant pathways (*p* < 0.05 & absolute Z score > 1). These formed four major functional groupings: (1) signaling pathways, (2) stress responses (3) proteostasis, and (4) the cell cycle, cell death and immune response (Fig. 5G). Intriguingly, the ATM and PTEN signaling pathways, which are associated with the regulation of longevity and the maintenance of cellular homeostasis under stress conditions [42–44], are significantly activated by hesperetin. Conversely, several signaling pathways, including HIF1α, ID1, HOTAIR, ERK/MAPK, and mTOR [45–49], which are associated with aging or senescence, are significantly inhibited by hesperetin (Fig. 5G). These transcriptomic results suggest that hesperetin is able to reduce cellular senescence and regulate redox homeostasis, which includes the response to oxidative stress, and the regulation of ROS metabolism, as well as oxidation–reduction (Fig. 5D and Additional file 1: Fig. S5D). Furthermore, the transcriptomic findings are also consistent with the phenotypic findings from hesperetin-treated HEK001 keratinocytes in terms of improved antioxidant capability and the promotion of mitochondrial function (Fig. 2E–G).

### Hesperetin activates longevity-associated regulators and suppresses the senescence-associated secretory phenotype

**Fig. 6:**
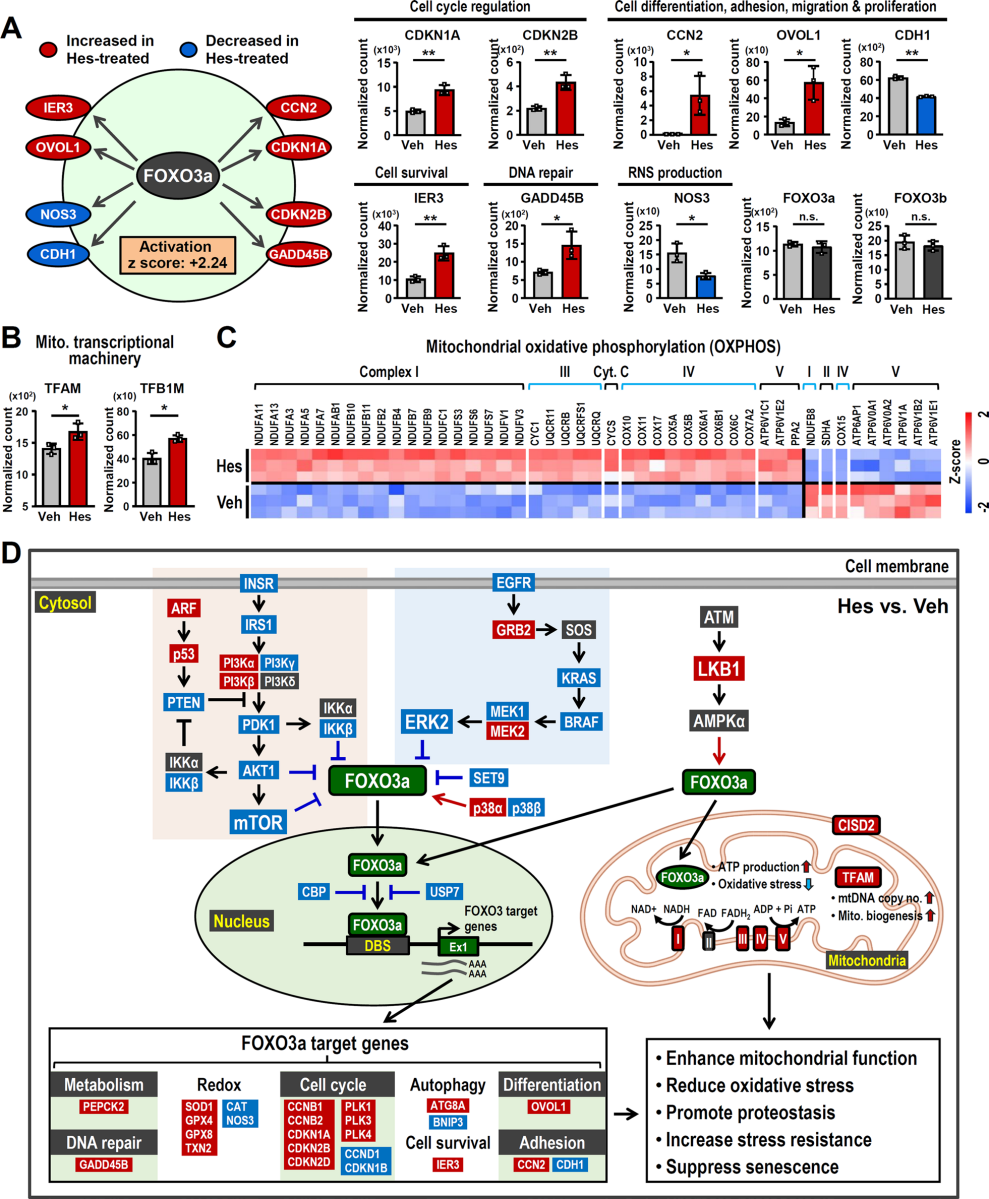
Hesperetin activates FOXO3a and its downstream target genes, and promoting mitochondrial function. **A** Significant activation of FOXO3a transcriptional signaling based on activation z-score (z-score > + 2.0 and *p*-value < 0.01) and IPA upstream regulator analysis of Hes-modulated DEGs in HEK001 keratinocytes. The mRNA levels of the DEGs are associated with FOXO3a-related transcriptional changes. The DEGs can be classified according to the different functions of the downstream target genes of FOXO3a. **B** The mRNA levels of TFAM and TFB1M in HEK001 keratinocytes. **C** A heatmap illustrating that hesperetin modulates the expression of OXPHOS-related genes in HEK001 keratinocytes. The criteria for the gene list in the heatmap is absolute fold change > 1.1 and *p* < 0.05 (Hes *vs* Veh). **D** A graphic summary of the DEGs and pathways associated with FOXO3a and modulated by hesperetin. Several upstream inhibitory pathways of FOXO3a are down-regulated by hesperetin. These include PI3K-AKT-mTOR, ERK-MAPK and other regulators (SET9, CBP and USP7) and these are known to suppress FOXO3a transcriptional activity by modulating its subcellular localization and DNA binding affinity. Conversely, ATM-LKB1-AMPK signaling and p38α are able to enhance FOXO3a activity via promoting its nuclear localization; both expression of LKB1 and p38α are increased by hesperetin. Additionally, AMPKα promotes an accumulation of FOXO3a in mitochondria in order to promote mitochondrial functions. Furthermore, ARF-p53-PTEN pathway indirectly activates FOXO3a via suppressing PI3K-AKT-mTOR signaling; ARF and p53 are found to be increased by hesperetin. Red boxes indicate upregulation and blue boxes indicate downregulation of mRNA levels. Red lines indicate activation and blue lines indicate suppression of FOXO3a activity. Data are presented as mean ± SD. *p < 0.05; **p < 0.005 by Student’s t test; not significant (n.s.). *DBS* DNA-binding sequence, *RNS* reactive nitrogen species, *SASP* senescence-associated secretory phenotype

To characterize the upstream regulators of these DEGs, we performed upstream analysis by IPA. A total of ten up-regulated and six down-regulated upstream regulators were identified (*p* < 0.01 and absolute Z score > 2; Additional file 1: Table S1). Interestingly, FOXM1 and FOXO3a, which are transcription factors involved in delaying aging and promoting longevity [50, 51], were identified as being significantly activated (Fig. 6A and Additional file 1: Fig. S6A). Furthermore, FOXO3a is believed to activate a variety of genes that are involved in cell proliferation, cell survival and DNA repair (Fig. 6A and Additional file 1: Fig. S6B). Similarly, FOXM1 has several downstream target genes, such as AURKB, BIRC5, BUB1B, PLK4 and CDK1 and these are also up-regulated. On the other hand, two immune response-related genes, IKBKB and NFKB2, were found to be down-regulated (Additional file 1: Fig. S6A). To validate the findings, we carried out a knockdown of FOXM1 in HEK001 keratinocytes before hesperetin treatment (Additional file 1: Fig. S6C). Intriguingly, hesperetin is unable to induce the expression of BUB1B and PLK4, two downstream target genes of FOXM1, in the FOXM1 KD keratinocytes (Additional file 1: Fig. S6D), indicating that FOXM1 is the upstream regulator of these genes under hesperetin treatment. To further characterize the upstream signaling of FOXO3a, we examined the mRNA levels of the upstream regulatory factors of FOXO3a, which include PI3K-AKT-mTOR, ERK and MAPK, as well as a number of other relevant regulators [50, 52, 53]. Interestingly, several inhibitory factors of FOXO3a are down-regulated by hesperetin. These include INSR, IRS1, PDK1, IKKβ, AKT1, and mTOR, which are part of the PI3K-AKT-mTOR signaling; furthermore, KRAS, BRAF and ERK2, which are part of ERK-MAPK signaling; as well as other regulators, such as SET9, CBP and USP7 are also affected (Additional file 1: Fig. S6E). Notably, TFAM (mitochondrial transcription factor A), which is essential for mitochondrial transcription and genome maintenance, and TFB1M (mitochondrial transcription factor B1), which is an auxiliary factor of mitochondrial transcription, are both significantly increased by hesperetin (Fig. 6B). Importantly, it is known that the activated form of FOXO3a is able to be translocated into both the nucleus and mitochondria; this allows transcriptional upregulation of genes involved in the functioning of mitochondria [52, 54]. Remarkably, after hesperetin treatment, a bunch of nucleus-encoded genes that make up Complex I, III, IV and V are significantly increased (Fig. 6C). In contrast to the above, interleukin-1α (IL-1α) and its downstream genes, which are associated with the immune response and SASP [55], are significantly down-regulated by hesperetin (Additional file 1: Fig. S6F). These findings suggest that hesperetin has both anti-inflammatory and anti-senescence effects. In fact, one of the IL-1α downstream target genes, MMP-1, is a UV-damage marker and, related to this, we have already shown that the protein level of MMP-1 is down-regulated by hesperetin under UVB radiation (Fig. 2I). In addition, several immune response and inflammation-related pathways are significantly enriched by hesperetin treatment, including TNF signaling, IKK/NF-κB signaling, the cellular response to TGF-β stimulus, and IL-17 signaling (Fig. 5E and Additional file 1: Fig. S5E and S5F). These results suggest that hesperetin exerts its anti-inflammatory effects via the modulation of multiple pro-inflammatory factors and signals. In summary, our transcriptomic and pathway analyses suggest that hesperetin is likely to activate FOXO3a activity by suppressing its upstream inhibitory signaling. This then attenuates the expression of SASP factors by, in part by suppressing IL-1α signaling, as well as by enhancing CISD2 expression. These changes exert beneficial effects whereby mitochondrial functioning is enhanced, oxidative stress is reduced, and the ability to respond to UVB-induced damage is increased. These effects then suppress senescence in HEK001 keratinocytes that were obtained from an elderly male human individual (Fig. 6D).

## Discussion

We have shown here that, in old mice, a CISD2 activator such as hesperetin, via an enhancement of CISD2 expression, is able to act as an effective regimen for slowing down skin aging and rejuvenating aged skin. Four significant findings can be pinpointed. **Firstly**, in human skin, CISD2 is mainly expressed in the proliferating keratinocytes present in the epidermal basal layer. It would seem that CISD2 is down-regulated in sun-exposed sites on the epidermis. **Secondly**, in HEK001 human keratinocytes from an older human subject, hesperetin enhances mitochondrial function and protects against ROS-induced oxidative stress; this comes about via an enhancement of CISD2 expression and occurs in a CISD2-dependent manner. Additionally, hesperetin alleviates UVB-induced cellular damage and suppresses MMP-1, a major indicator of UVB-induced damage in keratinocytes. **Thirdly**, transcriptomic analysis has revealed that hesperetin modulates a panel of DEGs associated with mitochondrial energy metabolism, redox homeostasis, keratinocyte function, and the immune response in order to attenuate senescence and promote cellular health. Intriguingly, hesperetin activates two known longevity-associated regulators, namely FOXO3a and FOXM1, as well as down-regulating SASP. **Finally**, in mouse skin, hesperetin enhances CISD2 expression, which in turn ameliorates UVB-induced photoaging; this occurs via a mechanism involving CISD2. Most strikingly, late-life treatment with hesperetin in old mice, starting at 21-month old and lasting for 5 months, is able to retard skin aging and even rejuvenate naturally aged skin. Thus, these results indicate that hesperetin, a CISD2 activator, is a promising candidate for the development of a regimen that will slow down skin aging and promote skin health.

### Enhancing CISD2 retards skin aging and rejuvenates aged skin

Intrinsic or chronological skin aging, which depends on the passage of time per se, is influenced by genetics, hormonal changes and various metabolic processes. The thickness of the epidermis, and dermis within skin, has been found to gradually decrease during aging [33]. A reduction in cell proliferative capacity and an accumulation of senescent cells represent the most noteworthy changes that occur during skin aging [1, 5]. In addition, a decrease in DNA repair capacity and a loss of telomere stability are also associated with skin aging [56]. It is generally believed that an increase in oxidative stress and DNA damage, as well as an accumulation of mitochondrial abnormalities, are likely to be the mechanism underlying intrinsic skin aging [4].

CISD2 protects cells against several aging hallmarks, including mitochondrial dysfunction, loss of proteostasis, cellular senescence, stem cell exhaustion and chronic inflammation [15, 57]. Importantly, a genetic elevation of CISD2 ameliorates such age-associated phenotypes [15, 58]. Here we translate genetic evidence into a pharmaceutical application using a CISD2 activator, namely, hesperetin, in order to restore aging skin. Our findings indeed demonstrate that the level of CISD2 in the skin at a late-life stage can be targeted pharmaceutically by hesperetin. As a result of enhanced CISD2 expression, there are improvements in the mitochondrial health of human keratinocytes obtained from an older human subject and a delay (or even rejuvenation) of aged skin in old mice.

Skin aging is a complex phenomenon influenced by a multitude of factors, these include well-recognized sunlight exposure, as well as other important contributions like ventilation and friction. Ventilation plays a pivotal role in maintaining the microenvironment of skin. Poor ventilation can disrupt various processes, potentially exacerbating skin conditions and thus accelerating the aging process [59]. Friction, whether from external sources or via repetitive mechanical stress, is another factor to consider. Friction can lead to alterations in skin elasticity and affect the extracellular matrix, which, over time, may contribute to changes in skin texture and resilience [60]. Overall, UV irradiation is probably the most detrimental extrinsic factor that damages skin and the result is skin photoaging. UV radiation triggers the generation of oxidative stress, inflammation, immunosuppression, apoptosis, and MMP production, all of which lead to the onset of photoaging [36]. UV-induced photoaging is directly linked to mitochondrial DNA depletion and mitochondrial dysfunction; these are accompanied by increased ROS production, and extracellular matrix degradation [8]. Strikingly, UBV exposure results in down-regulation of CISD2 with a concomitant increase in MMP-1, ROS and RNS production. Intriguingly, pharmacological up-regulation of CISD2 by hesperetin in mice reverses these adverse effects of UBV irradiation that are associated with skin damage.

### Clinical application of hesperetin to human subjects

Previously a clinical trial revealed that hesperetin is able to improve skin photo-aging in human subjects. The efficacy and safety of hesperetin, when used as a topical treatment of the skin, has been examined by a single-center clinical trial (ClinicalTrials.gov Identifier: NCT04015063) [61]. This study evaluated the efficacy of a skincare product (P29429-01, Orient EuroPharma Co., Ltd) that contains 0.1% hesperetin (derived from citrus peel) and 0.1% sodium cyclic lysophosphatidic acid (extracted from soybean), and examined the products effect on photo-aged skin (topical application twice a day for 12 weeks). A total of 35 female subjects (55.4 ± 5.75 years old) were enrolled. Their findings revealed that there is a significant decrease in the number of wrinkles, and a significant improvement in skin hydration and elasticity, both of which are characteristics associated with skin aging. Accordingly, topical application of a skincare product containing hesperetin is a safe and potent strategy for the enhancement of CISD2 expression and would seem to be an effective means of treating photo-aged skin.

### Potential mechanisms underlying the effects of hesperetin on aging skin

Previous studies using a variety of cell models have shown that hesperetin is able to affect many aging-associated signaling pathways, as well as suppressing several pro-inflammatory pathways [28, 62–64]. Consistently, our transcriptomic analysis revealed that hesperetin modulates multiple aging-related pathways in keratinocytes from an older person, such as the P53, MAPK, mTOR, HIF-1α, and TNFα signaling pathways. In addition, we found that hesperetin influences advanced glycation end products (AGE) and the receptor for the AGE (RAGE) pathway, which have been reported to be abnormally increased in several tissues during aging [65, 66]. Interestingly, studies from other groups have suggested that hesperetin is able to attenuate AGE generation and inhibit AGE-induced oxidative stress [67, 68]. The RAGE pathway, involving AGEs and their receptor RAGE, plays a significant role in the aging process, particularly in the context of skin health. Non-enzymatic glycation, which leads to the formation of AGEs, poses a threat to various important skin components [69]. AGEs can damage the skin by covalently binding to proteins. Moreover, it has been noted that RAGE expression in the skin varies with age and sun exposure [70]. In fetal skin, RAGE is primarily found in the upper epidermis and a few dermal endothelial cells. However, in aged and chronically sun-exposed skin, the RAGE distribution shifts to the deeper layers of the epidermis and the dermis, indicating their involvement in the aging of skin. This pathway likely contributes to the structural and functional changes seen in aged skin, including the development of wrinkles, loss of elasticity, and various altered cellular responses, making it a key factor in skin aging. Furthermore, the NF-kB pathway, which is enriched after hesperetin treatment, is a central mediator of the inflammatory response, regulating the expression of pro-inflammatory, immunomodulatory, and anti-apoptotic genes [71, 72]. These genes are part of the inflammatory cascade and have a multifaceted impact on the aging process of skin, which is characterized by wrinkles and reduced elasticity. Within the context of skin aging and various aging-related processes, the immune response-related genes IKBKB and NFKB2, which are integral to the NF-κB signaling pathway, play a pivotal role [73, 74]. Moreover, NF-κB signaling extends its influence to cellular senescence and apoptotic processes in various tissues, potentially impacting tissue function and regeneration during aging. Thus, hesperetin exerts its anti-aging, anti-oxidant, and anti-inflammation capabilities via the regulation of multiple signaling pathways.

A number of evolutionary conserved transcriptional factors have been identified as the regulators of longevity [75, 76]. FOXO3a has been recognized as one of two human pro-longevity genes and represents a major pharmaceutical target for the development of regimens that promote healthy longevity in an aging population [50]. FOXO3a, a transcriptional factor, maintains cellular homeostasis and extends lifespan via regulation of the expression of multiple downstream target genes that are involved in energy metabolism, redox status, mitochondrial functions, autophagy, stress resistance, cell proliferation, DNA repair, and telomere maintenance [52, 53, 77, 78]. Previous studies have indicated that FOXO3a participates in the maintenance of skin homeostasis and skin health [79, 80]. Therefore, activation of FOXO3a is a promising therapeutic strategy for reducing skin aging [50, 81]. In this study, FOXO3a was identified as being significantly activated by hesperetin and to be one of the upstream regulators of the DEGs associated with hesperetin treatment. Accordingly, it is likely that FOXO3a and its downstream target genes are involved in the molecular mechanism underlying the beneficial effects of hesperetin in relation to HEK001 human keratinocytes from an older human subject. Additionally, FOXM1, which is another transcription factor involved in delaying aging and promoting longevity [51], has been also identified as one of the upstream regulators of our identified DEGs. Moreover, it has been shown that, in primary neonatal human keratinocytes, FOXM1 is involved in the maintenance of a high cell proliferative potential, and the reduction of cellular senescence and oxidative stress [82].

Interestingly, the canonical forkhead motif, which is the putative DNA binding sequence of FOXO3a and FOXM1, is present within the promoter region (− 483 to − 476) of the human CISD2 gene. Notably, an age-dependent decline in FOXO3a and FOXM1 expression has been detected in several tissues of naturally aged mice [51, 83, 84]. This is similar to the observation that CISD2 is downregulated during aging. This, in turn, suggests that FOXO3a and FOXM1 maybe two of the upstream factors that modulate CISD2 transcription. As such, decreases in FOXO3a and FOXM1 expression are likely to lead to downregulation of CISD2. However, the connection between the activation of these two longevity-related transcriptional factors and CISD2 expression remains unproven. Accordingly, in the future, it will be of great importance to study if the transcription of the CISD2 gene is directly regulated by FOXO3a and FOXM1.

CISD2 is a versatile protein that has notable implications in both age-related diseases and cancers. Both genetic and pharmaceutical methods of increasing CISD2 levels have shown promise in relation to ameliorating age-associated diseases by enhancing mitochondrial function, reducing mitochondrial DNA damage, and improving Ca^2+^ regulation. Additionally, CISD2 overexpression has demonstrated to have a protective effect against Alzheimer’s disease by reducing neuronal death and inflammation in the mouse hippocampus. However, the role of CISD2 in cancer appears context-dependent, whereby it can in certain scenarios in animal studies function as a tumor suppressor and inhibit hepatocellular carcinoma development [15]. On the other hand, in other scenarios CISD2 can promote tumor cell growth and survival, potentially making it a target for the development of anticancer therapies. Future studies are required to increase our understanding the impact of CISD2 on signaling pathways in cancer cells. Nonetheless, the above underscores the versatile role of CISD2 in maintaining cellular homeostasis and emphasizes the complex relationship between CISD2 expression levels and health outcomes.

## Conclusion

Here we provide evidence for and demonstrate that the pharmacological elevation of CISD2 protein levels at a late-life stage is feasible and that such an increase attenuates both intrinsic aging of skin (i.e. aging via time per se) and extrinsic aging of skin (e.g. aging by sunlight and UV radiation). Hesperetin is the first compound that we have tested as a proof-of-concept for the hypothesis that a CISD2 activator has an anti-aging effect on the skin. Therefore, it is of great interest to begin to develop hesperetin either as a functional food or as a skincare product for topical treatment, with the aim of slowing down and/or rejuvenating aging skin via an enhancement of CISD2 expression and by the activation of various geroprotective transcriptional factors, such as the FOXO3a and FOXM1, which are believed to modulate various other longevity-related signaling pathways.

### Supplementary Information

**Additional file 1: Figure S1.** CISD2 is mainly expressed in the proliferating keratinocytes of the epidermis in normal human skin from an older person. Related to Fig. 1. **Figure S2.** Toxicity testing of different dosages of hesperetin against HEK001 human keratinocytes from an older person. Related to Fig. 2. **Figure S3.** Oral administration of hesperetin is able to ameliorate UVB-induced skin photoaging in WT mice. Related to Fig. 3. **Figure S4.** Hesperetin enhances Cisd2 expression in the skin of WT mice. Related to Fig. 4. **Figure S5.** Hesperetin modulated gene expression profiles using HEK001 human keratinocytes from an older person, These are related to proteostasis, cellular senescence, stress response and redox homeostasis. Related to Fig. 5. **Figure S6.** Hesperetin modulates the activity of FOXM1 and IL-1α, as well as the expression of FOXO3a downstream target genes in HEK001 keratinocytes. Related to Fig. 6. **Table S1.** Hesperetin-modulated changes in the upstream regulators and their downstream target genes in HEK001 keratinocytes.

## Data Availability

The datasets generated during and/or analyzed during the current study are available from the corresponding author upon reasonable request.

## Abbreviations

AGE: Advanced glycation end products
CISD2: CDGSH iron-sulfur domain-containing protein 2
DEGs: Differentially expressed genes
ER: Endoplasmic reticulum
FDR: False discovery rate
GO: Gene ontology
IHC: Immunohistochemistry
IL-1α: Interleukin-1α
IPA: Ingenuity pathway analysis
KD: Knockdown
KO: Knockout
MMPs: Matrix metalloproteinases
MMP-1: Matrix metalloproteinases-1
OCR: Oxygen consumption rate
PBS: Phosphate-buffered saline
RAGE: Receptor for the AGE
RNA-seq: RNA sequencing
RNS: Reactive nitrogen species
ROS: Reactive oxygen species
SASP: Senescence-associated secretory phenotype
SD: Standard deviation
shCISD2: CISD2 KD
TFAM: Mitochondrial transcription factor A
TFB1M: Mitochondrial transcription factor B1
TG: Transgenic
TMA: Tissue microarray
UV: Ultraviolet
UVA: Ultraviolet A
UVB: Ultraviolet B
WT: Wild-type
