# Supplementary material for: Hesperetin activates CISD2 to attenuate senescence in human keratinocytes from an older person and rejuvenates naturally aged skin in mice
Source: J Biomed Sci. 2024 Jan 23;31:15. doi: 10.1186/s12929-024-01005-w (PMC10807130; doi:10.1186/s12929-024-01005-w)
Supplement: Supplementary file 1 — Additional file 1: Figure S1. CISD2 is mainly expressed in the proliferating keratinocytes of the epidermis in normal human skin from an older person. Related to Fig. 1. Figure S2. Toxicity testing of different dosages of hesperetin against HEK001 human keratinocytes from an older person. Related to Fig. 2. Figure S3. Oral administration of hesperetin is able to ameliorate UVB-induced skin photoaging in WT mice. Related to Fig. 3. Figure S4. Hesperetin enhances Cisd2 expression in the skin of WT mice. Related to Fig. 4. Figure S5. Hesperetin modulated gene expression profiles using HEK001 human keratinocytes from an older person, These are related to proteostasis, cellular senescence, stress response and redox homeostasis. Related to Fig. 5. Figure S6. Hesperetin modulates the activity of FOXM1 and IL-1α, as well as the expression of FOXO3a downstream target genes in HEK001 keratinocytes. Related to Fig. 6. Table S1. Hesperetin-modulated changes in the upstream regulators and their downstream target genes in HEK001 keratinocytes. [file 12929_2024_1005_MOESM1_ESM.pdf]

Additional file 1

Figure S1 CISD2 is mainly expressed in the proliferating keratinocytes of the epidermis in normal human skin from an older person. Related to Figure 1.

Figure S2 Toxicity testing of different dosages of hesperetin against HEK001 human keratinocytes from an older person. Related to Figure 2.

Figure S3 Oral administration of hesperetin is able to ameliorate UVB-induced skin photoaging in WT mice. Related to Figure 3.

Figure S4 Hesperetin enhances Cisd2 expression in the skin of WT mice. Related to Figure 4.

Figure S5 Hesperetin modulated gene expression profiles using HEK001 human keratinocytes from an older person, These are related to proteostasis, cellular senescence, stress response and redox homeostasis. Related to Figure 5.

Figure S6 Hesperetin modulates the activity of FOXM1 and IL-1α, as well as the expression of FOXO3a downstream target genes in HEK001 keratinocytes. Related to Figure 6.

Table S1 Hesperetin-modulated changes in the upstream regulators and their downstream target genes in HEK001 keratinocytes

Figure S1

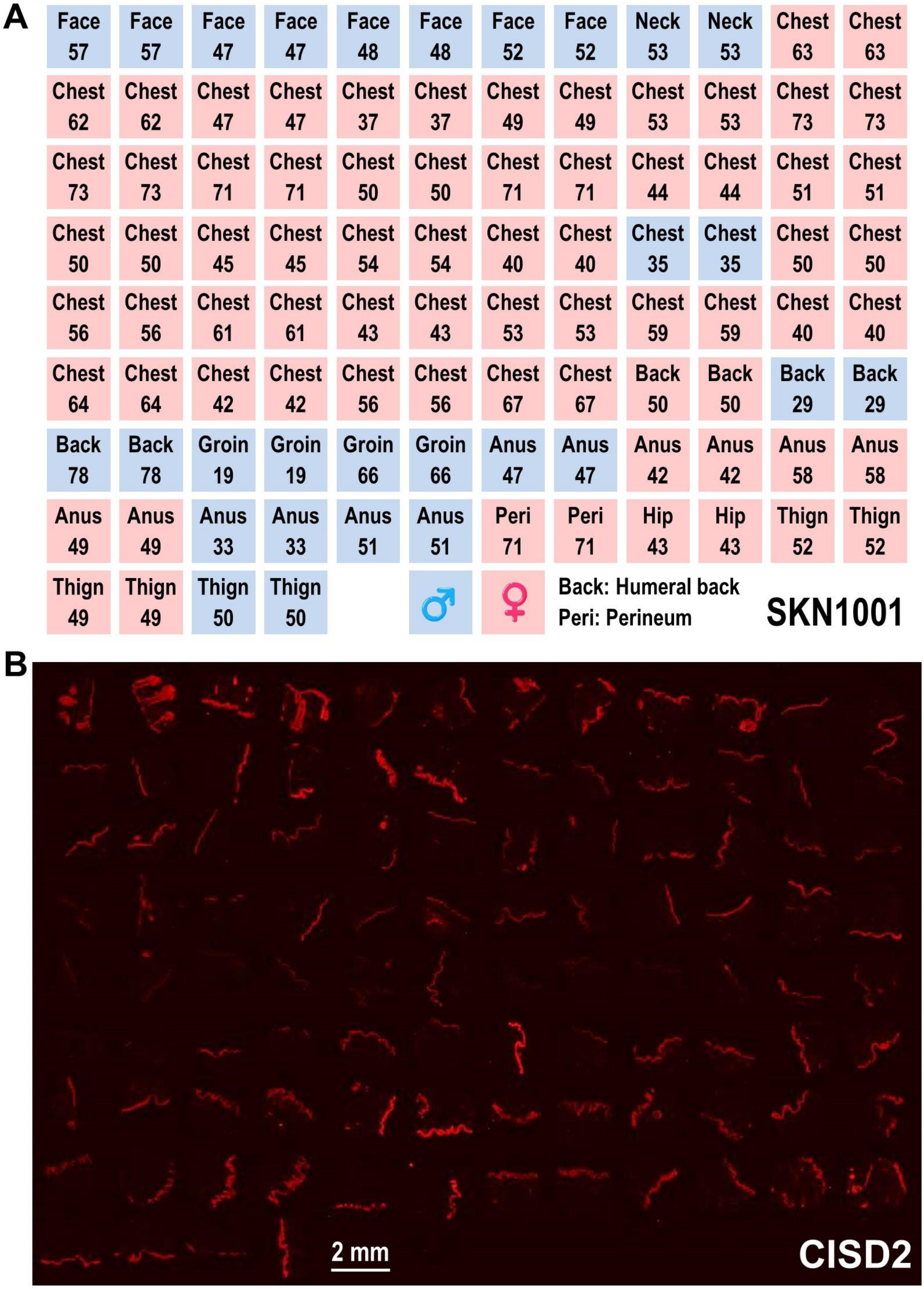
A
Face 57
Face 57
Face 47
Face 47
Face 48
Face 48
Face 52
Face 52
Neck 53
Neck 53
Chest 63
Chest 63
Chest 62
Chest 62
Chest 47
Chest 47
Chest 37
Chest 37
Chest 49
Chest 49
Chest 53
Chest 53
Chest 73
Chest 73
Chest 73
Chest 73
Chest 71
Chest 71
Chest 50
Chest 50
Chest 71
Chest 71
Chest 44
Chest 44
Chest 51
Chest 51
Chest 50
Chest 50
Chest 45
Chest 45
Chest 54
Chest 54
Chest 40
Chest 40
Chest 35
Chest 35
Chest 50
Chest 50
Chest 56
Chest 56
Chest 61
Chest 61
Chest 43
Chest 43
Chest 53
Chest 53
Chest 59
Chest 59
Chest 40
Chest 40
Chest 64
Chest 64
Chest 42
Chest 42
Chest 56
Chest 56
Chest 67
Chest 67
Back 50
Back 50
Back 29
Back 29
Back 78
Back 78
Groin 19
Groin 19
Groin 66
Groin 66
Anus 47
Anus 47
Anus 42
Anus 42
Anus 58
Anus 58
Anus 49
Anus 49
Anus 33
Anus 33
Anus 51
Anus 51
Peri 71
Peri 71
Hip 43
Hip 43
Thign 52
Thign 52
Thign 49
Thign 49
Thign 50
Thign 50
♂
♀
Back: Humeral back
Peri: Perineum
SKN1001
B
2 mm
CISD2

Figure S1 continued

C

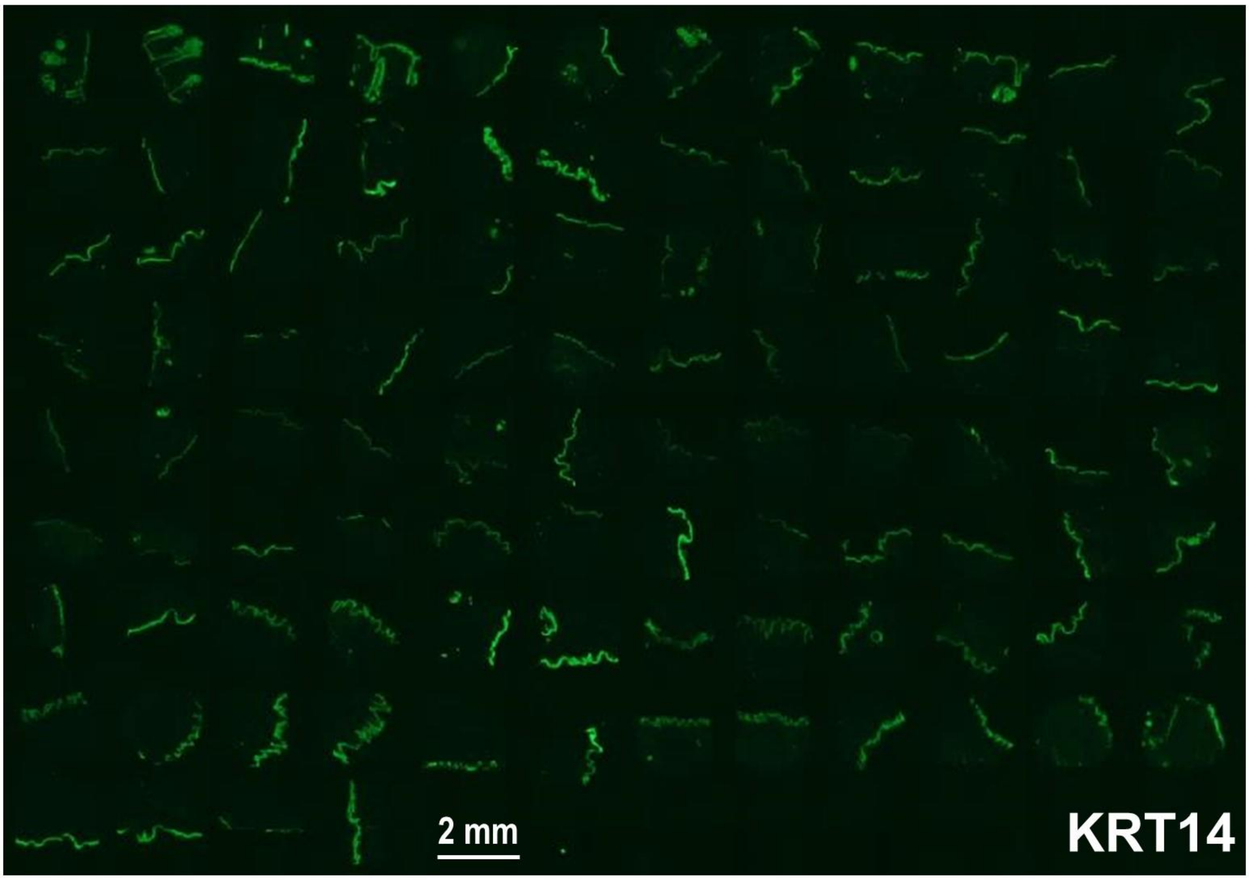

D

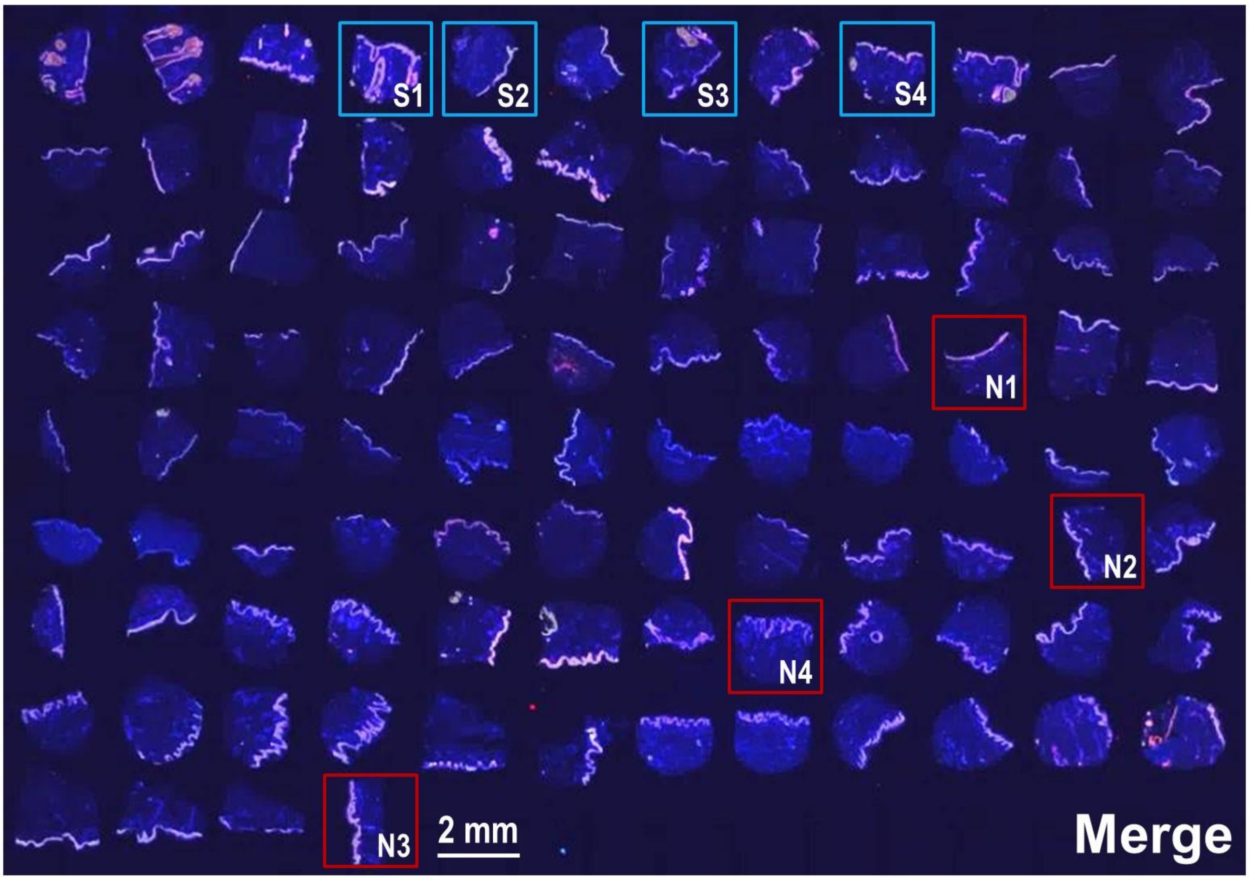

Figure S1 continued

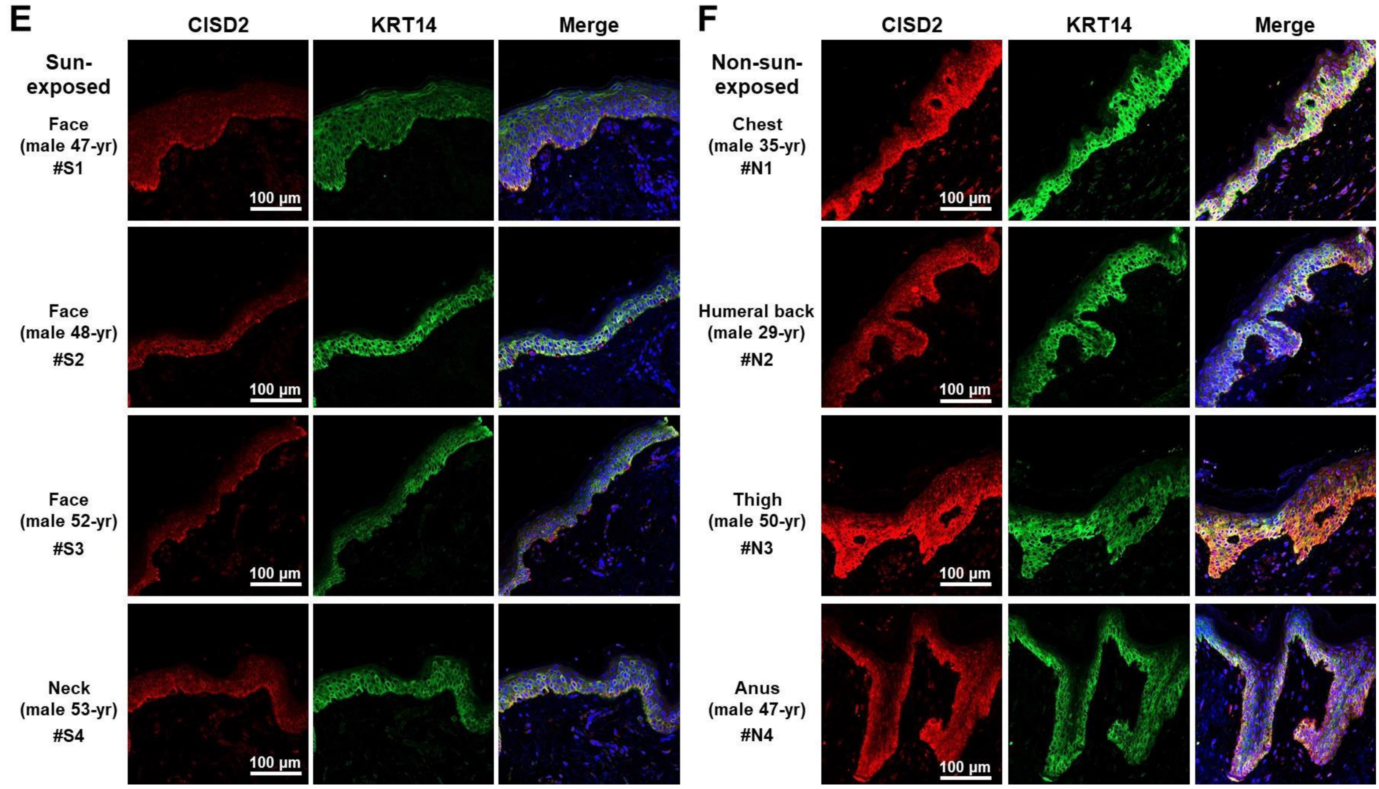

Figure S1. CISD2 is mainly expressed in the proliferating keratinocytes of the epidermis in normal human skin from an older person. Related to Figure 1.

(A) The age, sex and collection sites of the skin samples of the human tissues array SKN1001. **(B and C)** Fluorescent immunohistochemistry (IHC) staining of CISD2 (B) and KRT14 (a marker of proliferating keratinocytes in the epidermis) (C) in the normal human skin of the SKN1001 tissue array. **(D)** The merged IHC image of CISD2, KRT14 and DAPI in the SKN1001 tissue array. Blue boxes indicate the representative samples in panel E (#S1 to #S4). Red boxes indicate the representative samples in panel F (#N1 to #N4). Scale bars in (B-D), 2 mm. **(E)** Representative images of IHC staining of CISD2 and KRT14 from the sun-exposed sites (Face and Neck) of human skin. **(F)** Representative images of IHC staining of CISD2 and KRT14 from the non-sun-exposed sites, including chest, humeral back, thigh and anus of human skin. Scale bars in (E) and (F), 100 μm.

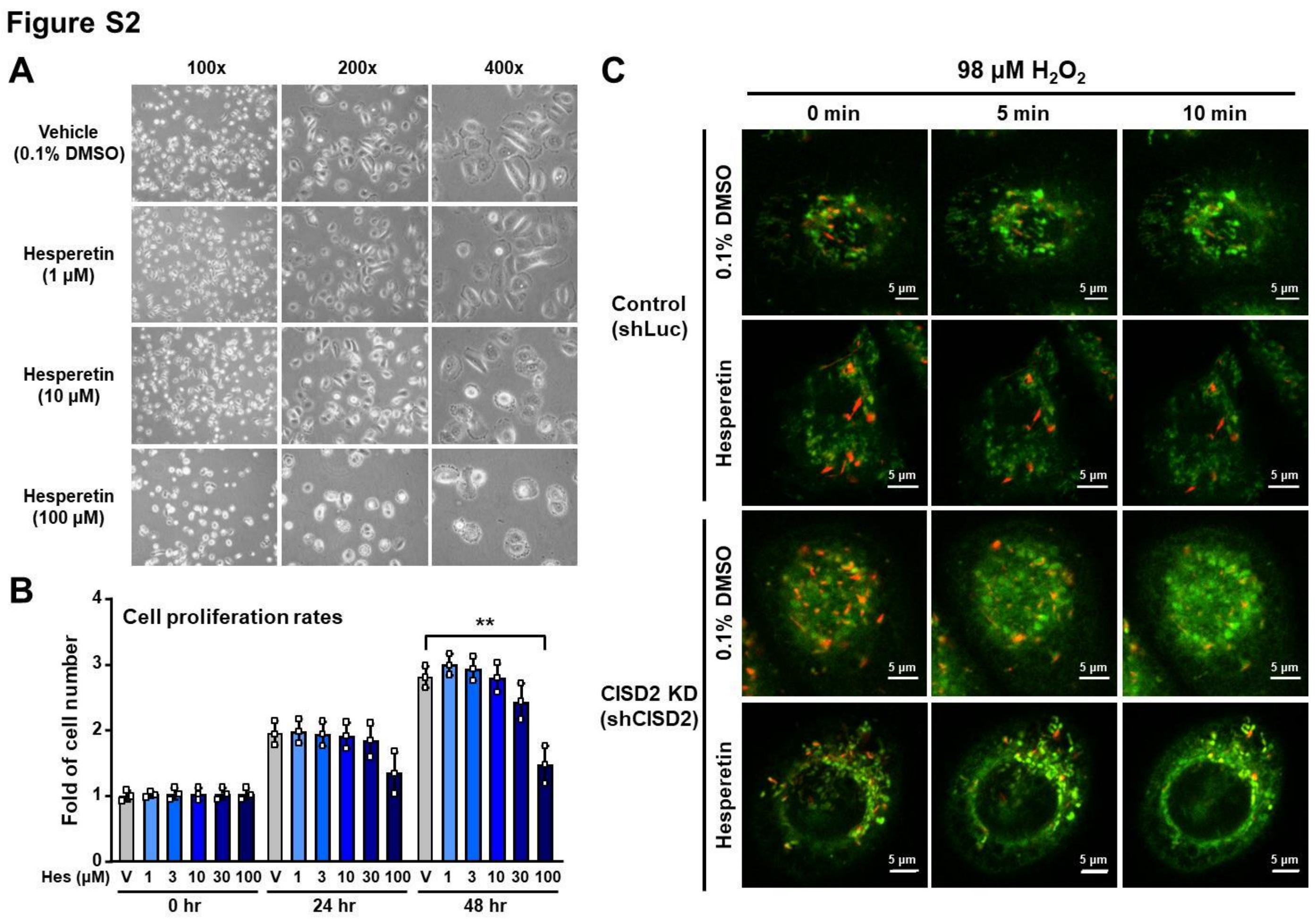

Figure S2. Toxicity testing of different dosages of hesperetin against HEK001 human keratinocytes from an older person. Related to Figure 2.

(A) Toxicity testing of different dosages of hesperetin against HEK001 keratinocytes. Cell morphology of the HEK001 keratinocytes after treatment with hesperetin at different dosages (1 µM, 10 µM and 100 µM) for 4 days. The cytotoxic effects, including a reduced cell density and a smaller cell size are present in the HEK001 keratinocytes at a high concentration (100 µM) of hesperetin treatment. **(B)** Analysis of cell proliferation rates after treatment of HEK001 keratinocytes with different concentrations of hesperetin (1-100 µM). The HEK001 keratinocytes were treated with different doses of hesperetin as indicated. Vehicle (V), 0.1% DMSO. **(C)** Representative images of JC-1 staining of the different groups of HEK001 keratinocytes. Hesperetin (10 µM) protects against oxidative stress-induced mitochondrial dysfunction. All experiments were performed and repeated three independent times as biological replicates using HEK001 keratinocytes. Data are presented as mean ± SD. The statistical analysis was performed by one-way ANOVA with Bonferroni multiple comparison test.

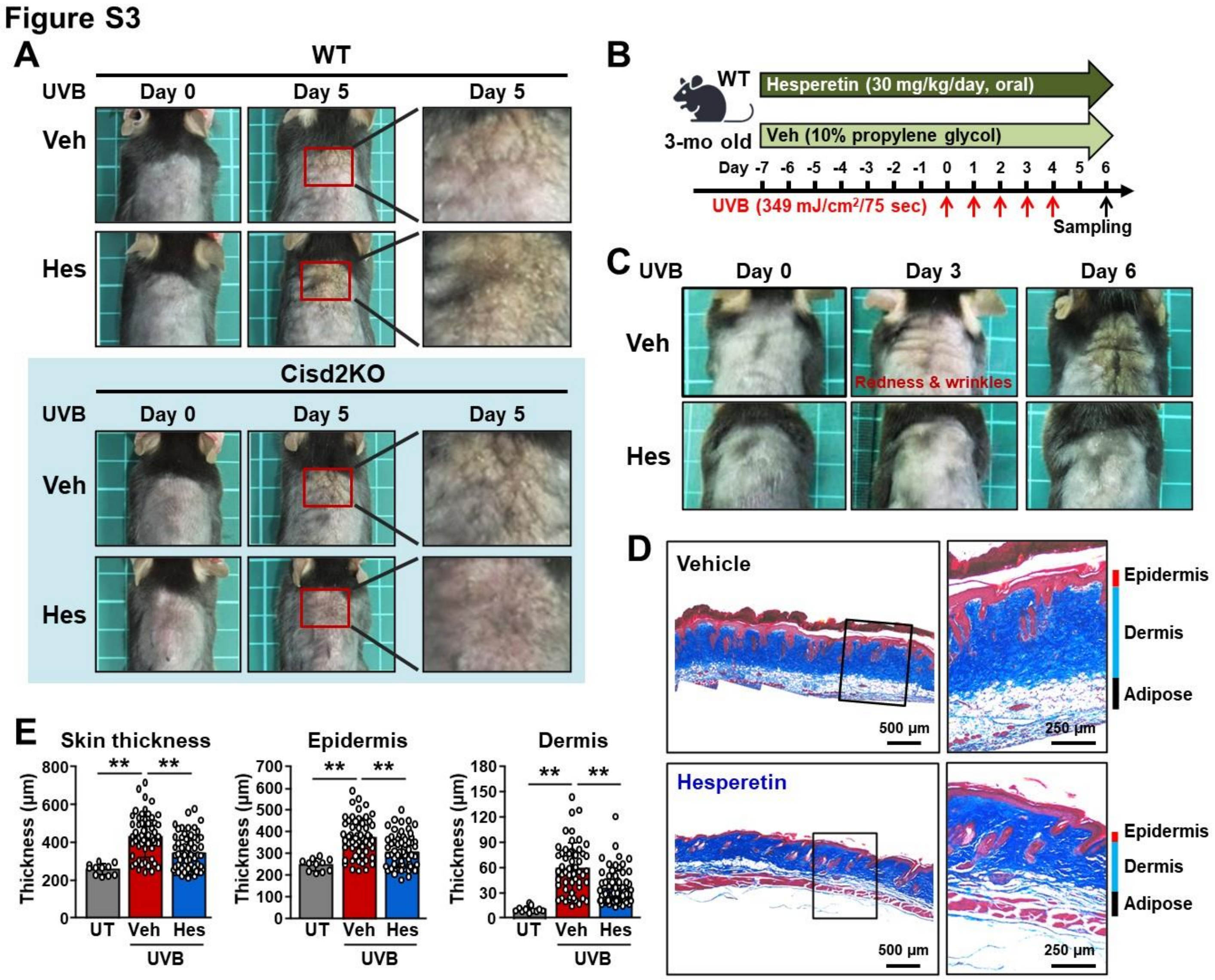

Figure S3. Oral administration of hesperetin is able to ameliorate UVB-induced skin photoaging in WT mice. Related to Figure 3.

(A) A gross view of the dorsal skin of Vehicle or Hesperetin treated WT and Cisd2KO mice before and after UVB exposure. **(B)** The protocol for oral treatment with hesperetin and its effect on UVB-induced skin damage in WT mice at 3-month old. The mice were pre-treated with hesperetin (30 mg/kg/day, oral administration) for 7 days, and then exposed to UVB (312 nm, 349 mJ/cm^2/75 seconds) light once a day for 5 days in a UVB box. The mice were sacrificed 6 days after the first UVB exposure. **(C)** A gross view of the dorsal skin of Vehicle or Hesperetin treated WT mice before and after UVB exposure. **(D)** Masson's trichrome staining of skin sections from the different groups of mice. UVB exposure significantly induces skin damage and increases skin thickness, which are the major characteristics of photoaging, while hesperetin treatment ameliorates UVB-induced skin damage. **(E)** Quantitation of the thickness of total skin layer, epidermal layer and dermal layer, in the skin of WT mice. Data are presented as mean ± SD. *$p < 0.05$; **$p < 0.005$ by one-way ANOVA with Bonferroni multiple comparison test; not significant (n.s.). UT, untreated.

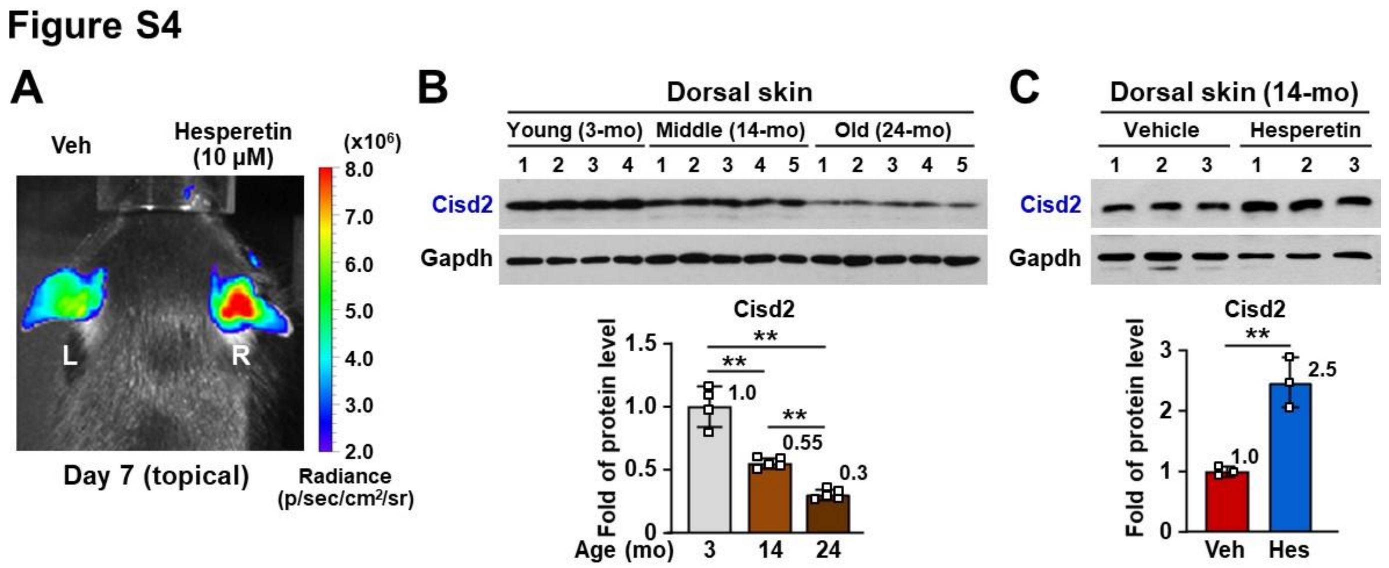

Figure S4. Hesperetin enhances Cisd2 expression in the skin of WT mice. Related to Figure 4.

(A) In vivo imaging system (IVIS) analysis of luciferase activity was examined in transgenic mice carrying the CISD2 BAC Luc reporter. Hesperetin stimulated luciferase reporter activity in the right ear of the CISD2 BAC reporter mice. Hesperetin (10 µM) and vehicle (10% glycerol with 5% menthol) were topically applied twice a day for 7 days to the right (R) and left (L) ears of mice at 3-month old, respectively. Luciferase activity was monitored for 7 days after hesperetin or Vehicle treatment. **(B)** Western blot analysis of Cisd2 protein levels revealed an age-dependent decline in Cisd2 protein expression in the skin from the young (3-month), mid-age (14-month) and old (24-month) wild-type C57BL/6 male mice (n = 4-5). **(C)** Hesperetin (10 mg/kg/day i.p. for 30 days) enhances by 2.5-fold the Cisd2 protein levels in the skin of middle-aged mice at 14-month old (n=3). The Cisd2 protein levels were determined by Western blot analysis and normalized using Gapdh. Data are presented as mean ± SD. *p < 0.05; **p < 0.005. In (B) the statistical analysis was performed by one-way ANOVA with Bonferroni multiple comparison test. In (C) the statistical analysis was performed by Student's t test.

Figure S5

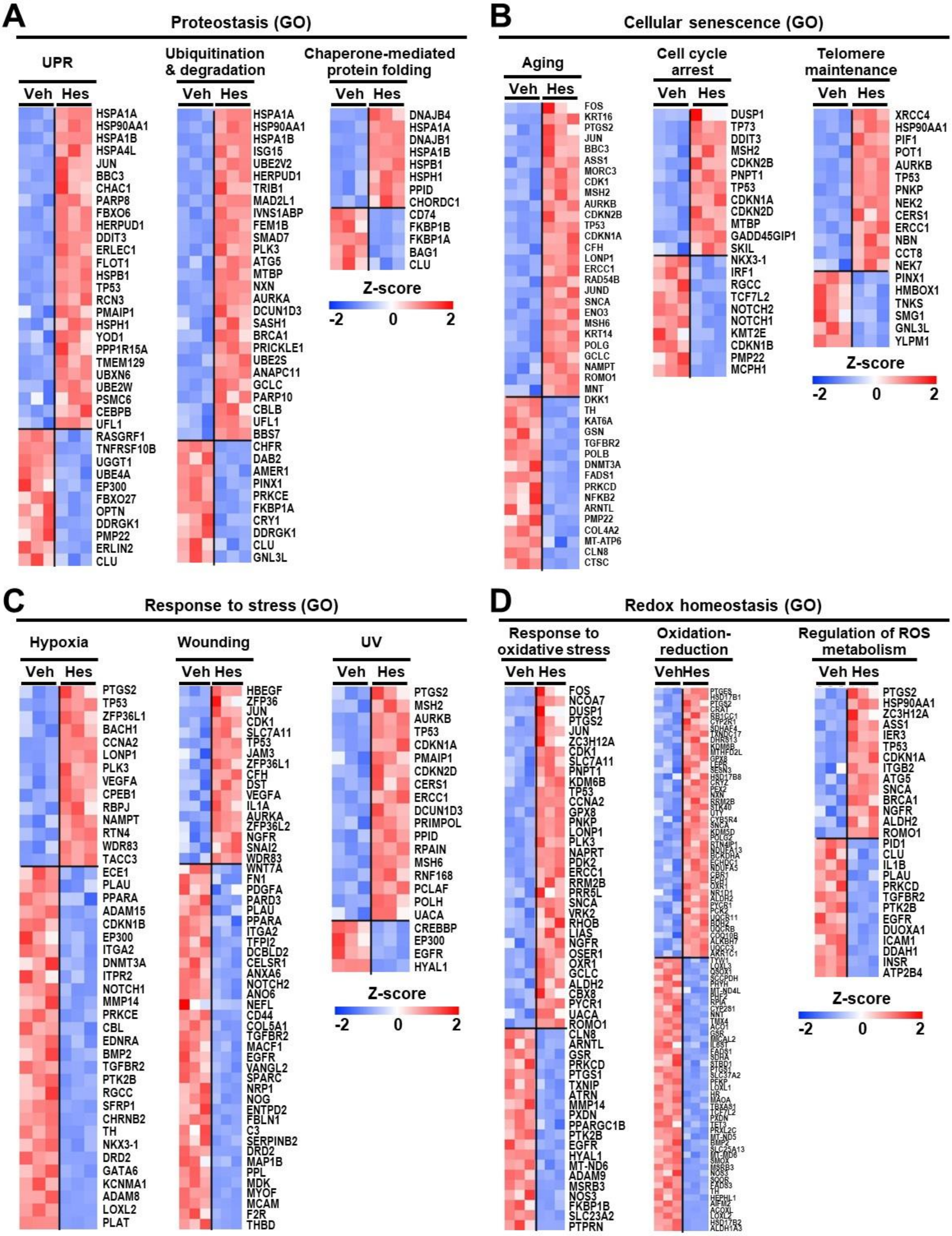
A
Proteostasis (GO)
UPR
Veh Hes
HSPA1A
HSP90AA1
HSPA1B
HSPA4L
JUN
BBC3
CHAC1
PARP8
FBXO6
HERPUD1
DDIT3
ERLEC1
FLOT1
HSPB1
TP53
RCN3
PMAIP1
HSPH1
YOD1
PPP1R15A
TMEM129
UBXN6
UBE2W
PSMC6
CEBPB
UFL1
RASGRF1
TNFRSF10B
UGGT1
UBE4A
EP300
FBXO27
OPTN
DDRGK1
PMP22
ERLIN2
CLU
Ubiquitination & degradation
Veh Hes
HSPA1A
HSP90AA1
HSPA1B
ISG15
UBE2V2
HERPUD1
TRIB1
MAD2L1
IVNS1ABP
FEM1B
SMAD7
PLK3
ATG5
MTBP
NXN
AURKA
DCUN1D3
SASH1
BRCA1
PRICKLE1
UBE2S
ANAPC11
GCLC
PARP10
CBLB
UFL1
BBS7
CHFR
DAB2
AMER1
PINX1
PRKCE
FKBP1A
CRY1
DDRGK1
CLU
GNL3L
Chaperone-mediated protein folding
Veh Hes
DNAJB4
HSPA1A
DNAJB1
HSPA1B
HSPB1
HSPH1
PPID
CHORDC1
CD74
FKBP1B
FKBP1A
BAG1
CLU
Z-score
-2 0 2
B
Cellular senescence (GO)
Aging
Veh Hes
FOS
KRT16
PTGS2
JUN
BBC3
ASS1
MORC3
CDK1
MSH2
AURKB
CDKN2B
TP53
CDKN1A
CFH
LONP1
ERCC1
RAD54B
JUND
SNCA
ENO3
MSH6
KRT14
POLG
GCLC
NAMPT
ROMO1
MNT
DKK1
TH
KAT6A
GSN
TGFBR2
POLB
DNMT3A
FADS1
PRKCD
NFKB2
ARNTL
PMP22
COL4A2
MT-ATP6
CLN8
CTSC
Cell cycle arrest
Veh Hes
DUSP1
TP73
DDIT3
MSH2
CDKN2B
PNPT1
TP53
CDKN1A
CDKN2D
MTBP
GADD45GIP1
SKIL
NKX3-1
IRF1
RGCC
TCF7L2
NOTCH2
NOTCH1
KMT2E
CDKN1B
PMP22
MCPH1
Telomere maintenance
Veh Hes
XRCC4
HSP90AA1
PIF1
POT1
AURKB
TP53
PNKP
NEK2
CERS1
ERCC1
NBN
CCT8
NEK7
PINX1
HMBOX1
TNKS
SMG1
GNL3L
YLPM1
Z-score
-2 0 2
C
Response to stress (GO)
Hypoxia
Veh Hes
PTGS2
TP53
ZFP36L1
BACH1
CCNA2
LONP1
PLK3
VEGFA
CPEB1
RBPJ
NAMPT
RTN4
WDR83
TACC3
ECE1
PLAU
PPARA
ADAM15
CDKN1B
EP300
ITGA2
DNMT3A
ITPR2
NOTCH1
MMP14
PRKCE
CBL
EDNRA
BMP2
TGFBR2
PTK2B
RGCC
SFRP1
CHRNB2
TH
NKX3-1
DRD2
GATA6
KCNMA1
ADAM8
LOXL2
PLAT
Wounding
Veh Hes
HBEGF
ZFP36
JUN
CDK1
SLC7A11
TP53
JAM3
ZFP36L1
CFH
DST
VEGFA
IL1A
AURKA
ZFP36L2
NGFR
SNAI2
WDR83
WNT7A
FN1
PDGFA
PARD3
PLAU
PPARA
ITGA2
TFPI2
DCBLD2
CELSR1
ANXA6
NOTCH2
ANO6
NEFL
CD44
COL5A1
TGFBR2
MACF1
EGFR
VANGL2
SPARC
NRP1
NOG
ENTPD2
FBLN1
C3
SERPINB2
DRD2
MAP1B
PPL
MDK
MYOF
MCAM
F2R
THBD
UV
Veh Hes
PTGS2
MSH2
AURKB
TP53
CDKN1A
PMAIP1
CDKN2D
CERS1
ERCC1
DCUN1D3
PRIMPOL
PPID
RPAIN
MSH6
RNF168
PCLAF
POLH
UACA
CREBBP
EP300
EGFR
HYAL1
Z-score
-2 0 2
D
Redox homeostasis (GO)
Response to oxidative stress
Veh Hes
FOS
NCOA7
DUSP1
PTGS2
JUN
ZC3H12A
CDK1
SLC7A11
PNPT1
KDM6B
TP53
CCNA2
GPX8
PNKP
LONP1
PLK3
NAPRT
PDK2
ERCC1
RRM2B
PRR5L
SNCA
VRK2
RHOB
LIAS
NGFR
OSER1
OXR1
GCLC
ALDH2
CBX8
PYCR1
UACA
ROMO1
CLN8
ARNTL
GSR
PRKCD
PTGS1
TXNIP
ATRN
MMP14
PXDN
PPARGC1B
PTK2B
EGFR
HYAL1
MT-ND6
ADAM9
MSRB3
NOS3
FKBP1B
SLC23A2
PTPRN
Oxidation-reduction
Veh Hes
Regulation of ROS metabolism
Veh Hes
PTGS2
HSP90AA1
ZC3H12A
ASS1
IER3
TP53
CDKN1A
ITGB2
ATG5
SNCA
BRCA1
NGFR
ALDH2
ROMO1
PID1
CLU
IL1B
PLAU
PRKCD
TGFBR2
PTK2B
EGFR
DUOXA1
ICAM1
DDAH1
INSR
ATP2B4
Z-score
-2 0 2

Figure S5 continued

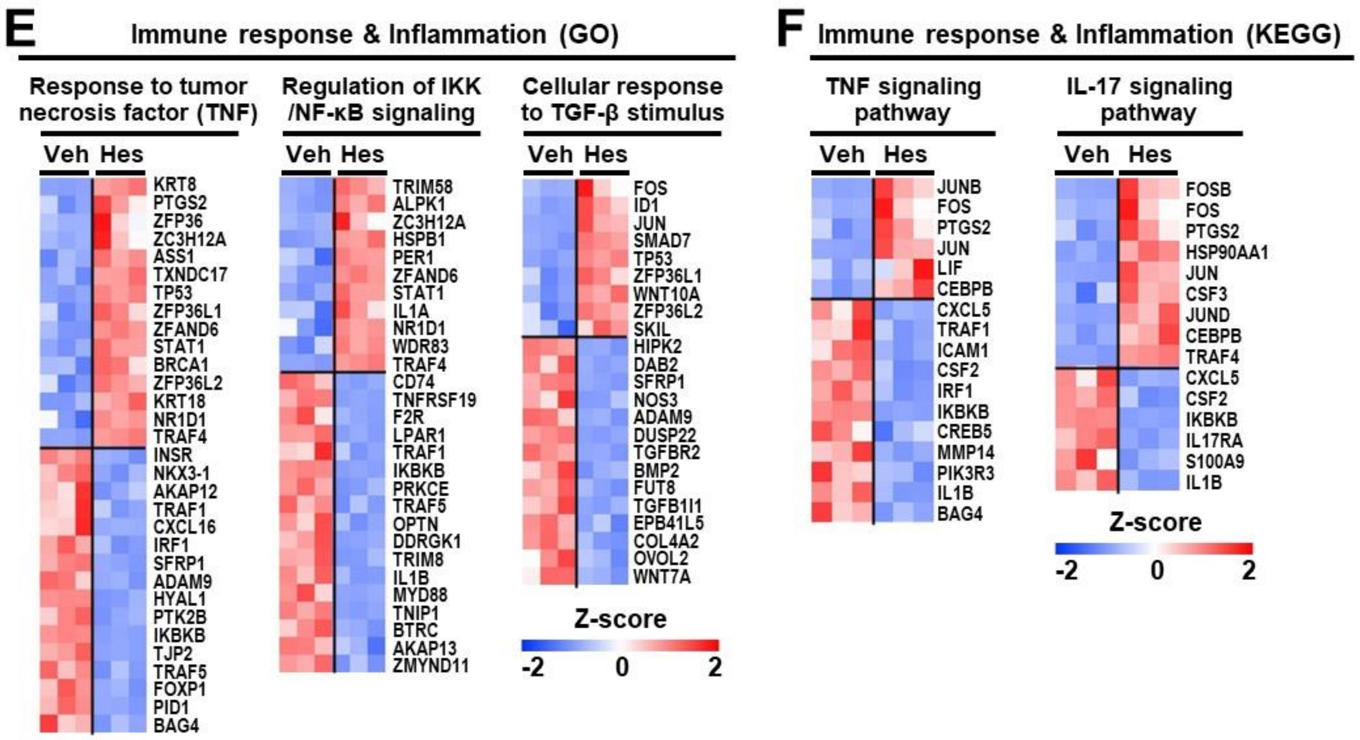

Figure S5. Hesperetin modulated gene expression profiles using HEK001 human keratinocytes from an older person, these are related to proteostasis, cellular senescence, stress response and redox homeostasis. Related to Figure 5.

(A) The heatmap pinpoints the proteostasis-related DEGs, including unfolded protein response (UPR), ubiquitination and proteasomal protein degradation, and chaperone-mediated protein folding, in HEK001 keratinocytes. **(B)** The heatmap pinpoints the cellular senescence-related DEGs, including aging, cell cycle arrest, and telomere maintenance in HEK001 keratinocytes. **(C)** The heatmap pinpoints the stress response-related DEGs, including response to UV, hypoxia, and wounding, in HEK001 keratinocytes. **(D)** The heatmap pinpoints the redox homeostasis-related DEGs, including oxidative stress response, oxidation-reduction, and regulation of reactive oxygen species (ROS) metabolism, in HEK001 keratinocytes. **(E and F)** The heatmap pinpoints the immune response and inflammation-related DEGs in HEK001 keratinocytes, including TNF signaling, IKK/NF-κB signaling, cellular response to TGF-β stimulus and IL-17 signaling.

Figure S6

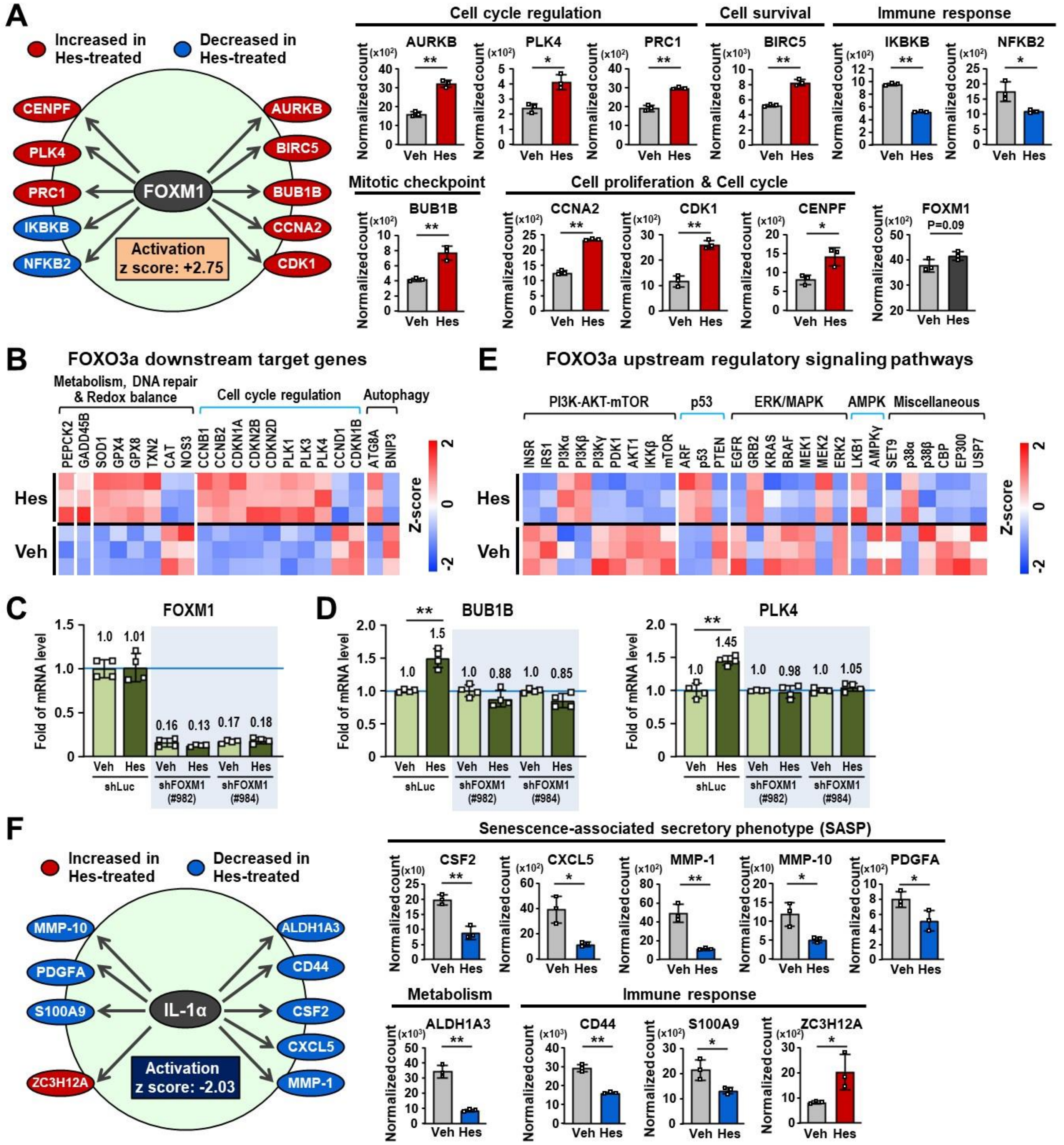

Figure S6. Hesperetin modulates the activity of FOXM1 and IL-1α, as well as the expression of FOXO3a downstream target genes in HEK001 keratinocytes. Related to Figure 6.

(A) Significant activation of FOXM1 transcriptional signaling is based on the activation z-score (z-score > +2.0 and p-value of overlap < 0.01) from IPA upstream regulator analysis of the Hesperetin modulated DEGs of HEK001 keratinocytes. The mRNA expression levels of the Hesperetin modulated DEGs are associated with

FOXM1-related transcriptional changes in HEK001 keratinocytes. The DEGs can be classified according to the different functions of the downstream target genes of FOXM1. These include cell cycle regulation, cell death prevention, mitotic checkpoint control, cell proliferation and immune response. **(B)** The heatmap shows that hesperetin modulates the expression of FOXO3a downstream target genes in HEK001 keratinocytes. **(C)** Real-time RT-qPCR analysis of FOXM1 mRNA levels revealed that >70% of the FOXM1 mRNA was knockdown (KD) by two independent FOXM1 shRNA clones (clone ID: TRCN0000273982 [#982] and TRCN0000273984 [#984]) treatments of HEK001 keratinocytes. The sequence of FOXM1 shRNA (Clone ID: TRCN0000273982; 5'-GCCAATCGTTCTCTGACAGAA-3'; Clone ID: TRCN0000273984; 5'-TTGCAGGGTGGTCCGTGTAAA-3'). The sequence of FOXM1 qPCR primers (FOXM1-F: 5'-TGCAGCTAGGGATGTGAATCTTC-3' and FOXM1-R: 5'-GGAGCCCAGTCCATCAGAACT-3'). **(D)** Real-time RT-qPCR analysis of FOXM1 downstream target genes (BUB1B and PLK4) mRNA levels in the Veh- or hesperetin-treated shLuc control and FOXM1 KD HEK001 keratinocytes. The sequence of qPCR primers (BUB1B-F: 5'-GAAGCTGAGCCCAATTATTG-3' and BUB1B-R: 5'-GAGTAGGGTTTTCTGAAGTC-3'; PLK4-F: 5'-GACACCTCAGACTGAAACCGTAC-3' and PLK4-R: 5'-GTCCTTCTGCAAATCTGGATGGC-3'). The mRNA levels by qPCR analysis were normalized against HPRT1. **(E)** The heatmap shows that hesperetin modulates the expression pattern of genes in various signaling pathways, associated with regulation of FOXO3a transcriptional activity in HEK001 keratinocytes. (D) Significant inhibition of IL1α cytokine signaling (z-score < -2.0 and p-value of overlap < 0.01) occurs in the Hesperetin modulated DEGs of HEK001 keratinocytes. The DEGs can be classified according to the different functions of the downstream target genes of IL1α. This includes senescence-associated secretory phenotype (SASP), metabolism and immune response. The DEGs were analyzed by IPA upstream analysis. The criteria for the gene list in the heatmaps are absolute fold change > 1.1 and $p < 0.05$ (Hes vs Veh). The data are presented as mean ± SD. *$p < 0.05$; **$p < 0.005$; not significant (n.s.). In (A) and (F) the statistical analysis was performed by Student's t test. In (C) and (D) the statistical analysis was performed by one-way ANOVA with Bonferroni multiple comparison test.

Table S1. Hesperetin-modulated changes in the upstream regulators and their downstream target genes in HEK001 keratinocytes.

Upstream Regulator	Molecule Type	Predicted Activation State	Activation z-score	p-value	Target molecules in dataset
CKAP2L	Other	Activated	4.000	3.51E-07	AURKB,BIRC5,CCNB2,CDK1,CENPF,ERCC6L,KIF23,KIF2C,MAD2L1,NCAPG,NDC80,NEK2,NUF2,PLK4,SPC24,SPC25
AREG	Growth factor	Activated	2.892	1.21E-04	AURKB,BIRC5,C3,CCNA2,CCNB2,CENPF,IFI6,KIF14,MMP15,PLAU,PRC1,PTAFR,SLC36A1,TOP2A
**FOXM1**	Transcription regulator	Activated	2.754	3.79E-05	AURKB,BIRC5,BUB1B,CCNA2,CDK1,CENPF,IKBKB,NFKB2,PLK4,PRC1
Interferon-α	Group	Activated	2.433	1.95E-03	IFIH1,IFIT1,ISG15,MX1,OAS1,STAT1
**FOXO4**	Transcription regulator	Activated	2.429	4.66E-04	CCN2,CDKN1A,CDKN2B,GADD45B,IER3,OVOL1
SMAD4	Transcription regulator	Activated	2.343	5.14E-03	CCN2,CDKN1A,CDKN2B,DLX3,EPB41L5,GADD45B,IER3,OVOL1,SNAI2,VEGFA
EPHA2	Kinase	Activated	2.335	4.64E-07	CD274,DUSP1,FOS,FOSB,IER3,IL1B,JUN,JUNB,NR4A1,ZFP36
**FOXO3**	Transcription regulator	Activated	2.239	5.66E-04	CCN2,CDH1,CDKN1A,CDKN2B,GADD45B,IER3,NOS3,OVOL1
NEUROG1	Transcription regulator	Activated	2.138	5.48E-05	AMIGO2,ASS1,C3,CD82,CEMIP,CFH,FN1,GFPT2,LRIG1,NOG,PXDN,SLC43A3,SPOCK1,SQOR
Notch	Group	Activated	2.013	4.24E-03	BIRC5,CDKN1A,CDKN2B,DUSP1,GADD45B,ID1,NFATC1,TAGLN
STAG2	Other	Inhibited	-2.688	2.63E-07	CD274,DDX60,DHX58,IFI44L,IFIH1,IRF7,IRF9,ISG15,ISG20,LGALS3BP,OAS1,SAMD9,SP110,UBE2L6
KDM5B	Transcription regulator	Inhibited	-2.360	1.38E-10	AURKA,BRCA1,BUB1B,CDCA3,CDK1,DDIT3,DLGAP5,EPB41L1,FABP5,HMMR,HSD17B8,ISG15,IVNS1ABP,KIF2C,LGALS3BP,MCAM,MT1E,NDC80,NEDD9,NMB,OSER1,POLB,PSD3,SAT1,SCNN1A,SMOX,TOP2A,TTK
IgG	Complex	Inhibited	-2.255	1.71E-09	CDKN1A,CEBPB,CRABP2,CTSC,DUSP1,FABP5,HSPB1,IER2,IFITM3,IL1B,IL1RN,ISG15,JUND,KRT10,KRT15,KRT16,KRT18,LDLR,LGALS7/LGALS7B,PIM1,PPL,PPP1R15A,RND3,SLC2A3,TRIM16,ZFP36
ATF3	Transcription regulator	Inhibited	-2.236	4.14E-04	AURKA,AURKB,CDK1,GSN,NEK2
LONP1	Peptidase	Inhibited	-2.236	6.56E-03	ASNS,CHAC1,MT-ATP6,MT-ND5,MT-ND6
**IL1α**	Cytokine	Inhibited	-2.028	5.90E-03	ALDH1A3,CD44,CSF2,CXCL5,IL1A,MMP-1,MMP-10,PDGFA,S100A9,ZC3H12A
